# Doping of Colloidal Nanocrystals for Optimizing Interfacial Charge Transfer: A Double-Edged Sword

**DOI:** 10.1021/jacs.4c06110

**Published:** 2024-08-27

**Authors:** Sheng He, Anji Ni, Sara T. Gebre, Rui Hang, James R. McBride, Alexey L. Kaledin, Wenxing Yang, Tianquan Lian

**Affiliations:** † Department of Chemistry, Emory University, 1515 Dickey Drive Northeast, Atlanta, Georgia 30322, United States; ‡ Department of Chemistry, The Vanderbilt Institute of Nanoscale Science and Engineering, Vanderbilt University, Nashville, Tennessee 37235, United States; § The Cherry L. Emerson Center for Scientific Computation, Emory University, 1515 Dickey Drive Northeast, Atlanta, Georgia 30322, United States

## Abstract

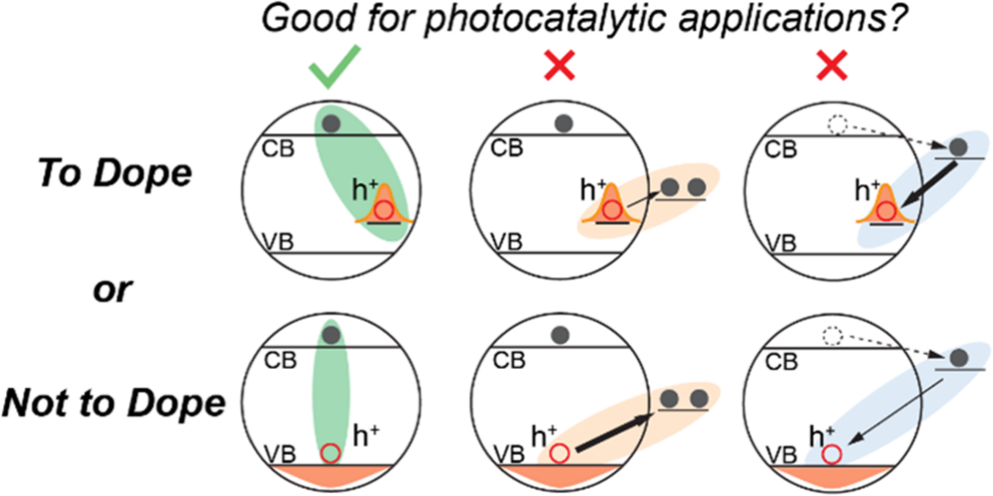

Doping of colloidal nanocrystals offers versatile ways to improve their optoelectronic properties, with potential applications in photocatalysis and photovoltaics. However, the precise role of dopants on the interfacial charge transfer properties of nanocrystals remains poorly understood. Here, we use a Cu-doped InP@ZnSe quantum dot as a model system to investigate the dopant effects on both the intrinsic photophysics and their interfacial charge transfer by combining time-resolved transient absorption and photoluminescent spectroscopic methods. Our results revealed that the Cu dopant can cause the generation of the self-trapped exciton, which prolongs the exciton lifetime from 48.3 ± 1.7 to 369.0 ± 4.3 ns, facilitating efficient charge separation to slow electron and hole acceptors. However, hole localization into the Cu site alters their energetic levels, slowing hole transfer and accelerating charge recombination loss. This double-edged sword role of dopants in charge transfer properties is important in the future design of nanocrystals for their optoelectronic and photocatalytic applications.

## Introduction

Colloidal nanocrystals (NCs) have been shown to have potential applications in many fields (e.g., in photocatalysis,^1,2^ photovoltaics,^3,4^ and photodetectors^5,6^), and improving the performance of these devices requires understanding and controlling their interfacial charge transfer properties. For photocatalytic applications, the photogenerated excitons in NCs should be long-lived enough to ensure the effective transfer of conduction band electrons (valence band holes) to the catalytic sites to drive reduction (oxidation) reactions.^1,7^ The back-recombination of electrons and holes in the charge-separated state should be minimized to reduce loss.^8^ Although in recent years, various approaches have been developed to improve the overall charge separation properties of NCs, including the use of colloidal heterostructures^8^ and surface engineering,^9−11^ achieving a long-lived charge-separated state is still a challenge because of the slow rates of many solar-fuel-forming reactions. Doping of NCs has been known to impact their photophysical properties.^12−19^ Small amounts of Ag dopants have been shown to improve the photoluminescence (PL) of CdSe quantum dots (QDs) due to accelerated radiative decay of electrons and holes.^14^ More recently, doped NCs have also been used in photoelectrochemical conversion. For example, addition of Cu into InP@ZnS NCs has been reported to enhance the solar-to-H_2_ conversion efficiency.^20,21^ However, it has also been reported that dopants can lower the photoelectrochemical conversion performances of NCs.^22,23^ These apparently conflicting dopant effects may be caused by the complexity of the overall photochemical light-to-fuel conversion process, which is comprised of multiple competing elementary steps, such as exciton recombination, forward electron transfer, hole extraction, and backward electron transfers.^7,24^ Despite its importance, the dopant impact on these elementary charge transfer steps, especially on charge transfers directly involving the dopant state, however, has not been systematically investigated.

**Scheme 1:**
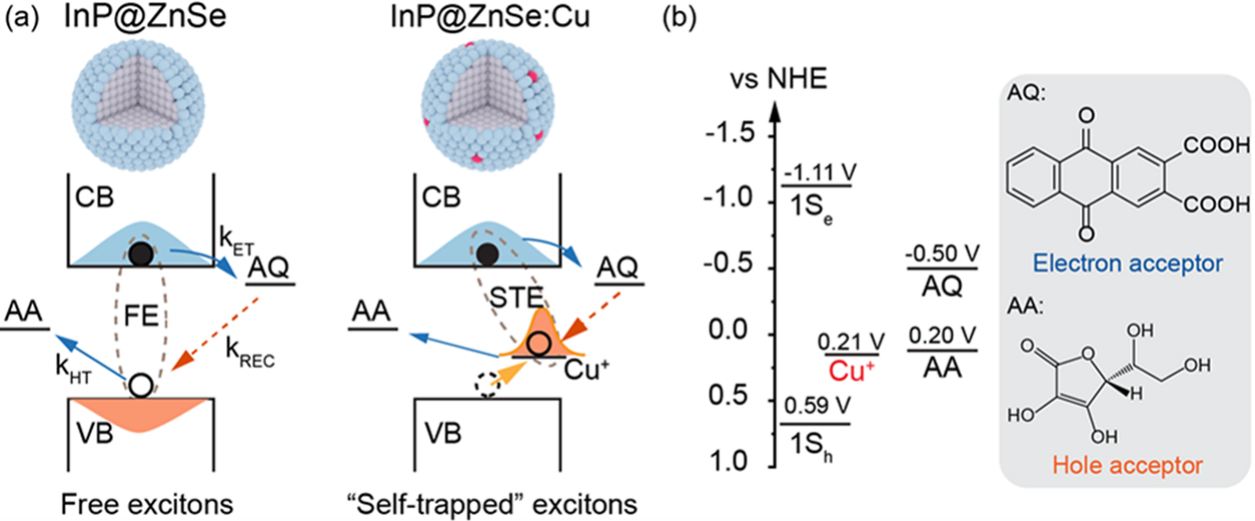
(a) Schematic of Free and Self-Trapped Excitons in InP@ZnSe and Cu-Doped InP@ZnSe QDs and the Key Charge Transfer Processes; (b) Energetic Levels of Relevant States in the QDs and Charge Acceptors versus the Normal Hydrogen Electrode (NHE) *k*_ET_ and *k*_HT_ are the electron/hole transfer (blue solid arrows) from QDs to the corresponding acceptors. *k*_REC_ represents the recombination (orange dashed arrows) of the electron in the reduced electron acceptor and the holes in QDs. Gray dashed lines mark the corresponding free excitons (FE) and self-trapped excitons (STE) discussed below. The energetic levels in QDs are estimated from the effective mass model (Section S10 and Table S6). The 1S_e_ and 1S_h_ levels represent the band edge electron and hole energy in the presence of exciton binding energy, respectively. Anthraquinone-2,3-dicarboxylic acid (AQ) and ascorbic acid (AA) are used as electron and hole acceptors to probe the impact of Cu dopants on interfacial charge transfers of InP@ZnSe QDs.

In this work, utilizing Cu-doped InP@ZnSe core/shell QDs as a model system, we investigate the effect of the Cu dopant on both the intrinsic photophysics of InP QDs and the elemental electron transfer (ET) and hole transfer (HT) steps of QD-acceptor complexes (Scheme 1a). InP QDs are chosen due to their promising applications in photocatalytic and optoelectronic technologies,^21,25−28^ with much less toxicity than Cd- or Pb-based NCs. Multiple electron and hole acceptors were employed as a probe of the interfacial charge transfer properties to provide a generally applicable conclusion from these studies (Scheme 1b). Our results show that the “self-trapped” exciton in Cu-doped InP QDs is significantly longer-lived than the band edge (or “free”) excitons, beneficial for achieving efficient interfacial charge separation in QD-acceptor complexes. However, localization of holes into Cu centers significantly reduces the hole extraction driving force and slows the HT process. Moreover, we also observed that after ET to electron acceptors, the Cu-doped QDs show a much faster recombination dynamics with the self-trapped holes than the hole in the valence band in undoped QDs, reducing the lifetime of the charge-separated state. These results reveal the important competing effects that should be considered when using dopants as a strategy to improve the charge transfer properties of NCs.

## Results

### Sample Preparation and Characterization

The InP@ZnSe core/shell QDs and Cu-doped InP@ZnSe QDs (InP@ZnSe:Cu) used in the present study were prepared according to reported procedures.^20,29^ Briefly, the InP core was first synthesized, followed by ZnSe shell growth using the successive ionic layer adsorption and reaction (SILAR) method.^30,31^ The shell growth was necessary to ensure the stability of the InP QDs. In both samples, the SILAR processes were repeated three times to grow ca. 2–3 monolayers of the ZnSe shell. For the InP@ZnSe:Cu QDs, Cu dopants were introduced after the first SILAR growth of ZnSe and prior to the second and third SILAR growth of ZnSe. Such growth of dopants into the shells of nanocrystals allows better control of the dopant location inside the nanocrystals^32,33^ and was recently reported to result in InP QDs with better photocatalytic performance.^20,21^

Transmission electron microscopy measurements (Figure S1) confirmed that InP@ZnSe and InP@ZnSe:Cu QDs have similar size and dispersity (4.1 ± 0.5 and 3.8 ± 0.8 nm, respectively); this ensures that the difference in the photophysics in these QDs is mainly caused by Cu doping and not QD size variations. Inductively coupled plasma mass spectrometry measurements reveal a Cu:In ratio of 5.7(±0.9):100 in the InP@ZnSe:Cu QDs, corresponding to 10.4 ± 1.6 Cu atoms per QD. Note that we also tested other InP@ZnSe:Cu samples with Cu amounts of ∼7 and ∼18 per QD, which all show qualitatively similar results as reported below and will be discussed later.

**Figure 1:**
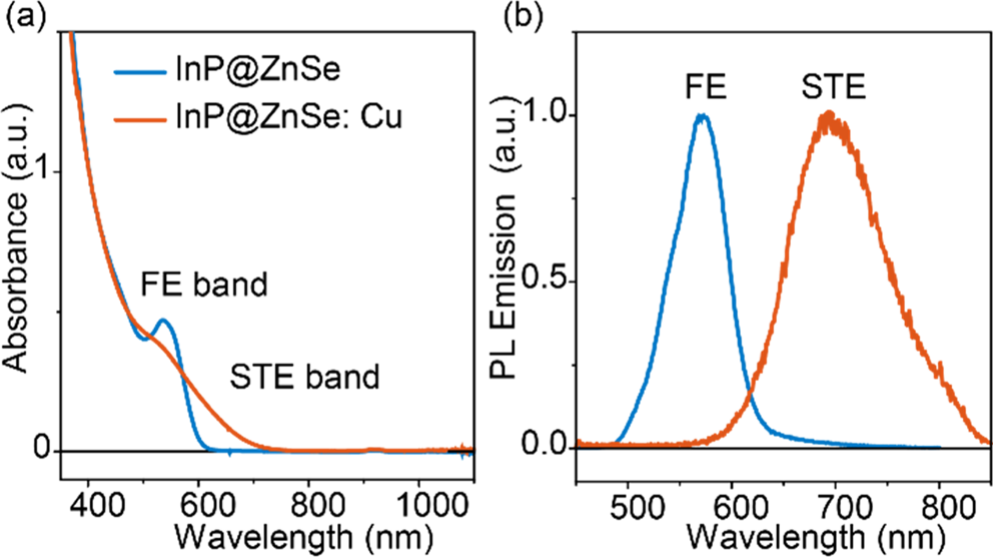
UV–vis absorption (a) and PL emission spectra (b) of InP@ZnSe and InP@ZnSe:Cu dispersed in hexane. Excitation wavelength of PL measurements: 400 nm.

The static absorption spectrum of InP@ZnSe QDs dispersed in hexane (Figure 1a) shows a band edge exciton (or free exciton) absorption at 540 nm (named the FE band). A similar absorption band is also observed in InP@ZnSe:Cu QDs, but with a much-reduced amplitude, accompanied by a new absorption band extending to ∼710 nm. This absorption is attributed to the transition between the Cu dopant and the conduction band of InP to form a self-trapped exciton (named as STE band hereafter), following a previous assignment of a similar feature in Cu-doped QDs.^18,34^ Photoexcitation of InP@ZnSe at 400 nm results in a photoluminescence (PL) band centered at 572 nm (Figure 1b), corresponding to the band edge exciton emission. However, photoexcitation of InP@ZnSe:Cu QDs at 400 nm results in negligible band edge emission but a new emission peak at 693 nm. This observation suggests the ultrafast trapping of photogenerated holes from the valence band into the Cu site, which subsequently recombine with the conduction band electron (see discussions below).^20,21,34^ From the shifts of PL between FE and STE states, the energetic level of the Cu dopant is estimated to be ∼0.38 eV above the 1S_h_ level of the InP core, assuming similar stoke shifts of both bands, consistent with the reported energy offset of Cu-doped ZnSe nanocrystals (∼0.40 eV).^35^

### Exciton Dynamics of Doped QDs

**Figure 2:**
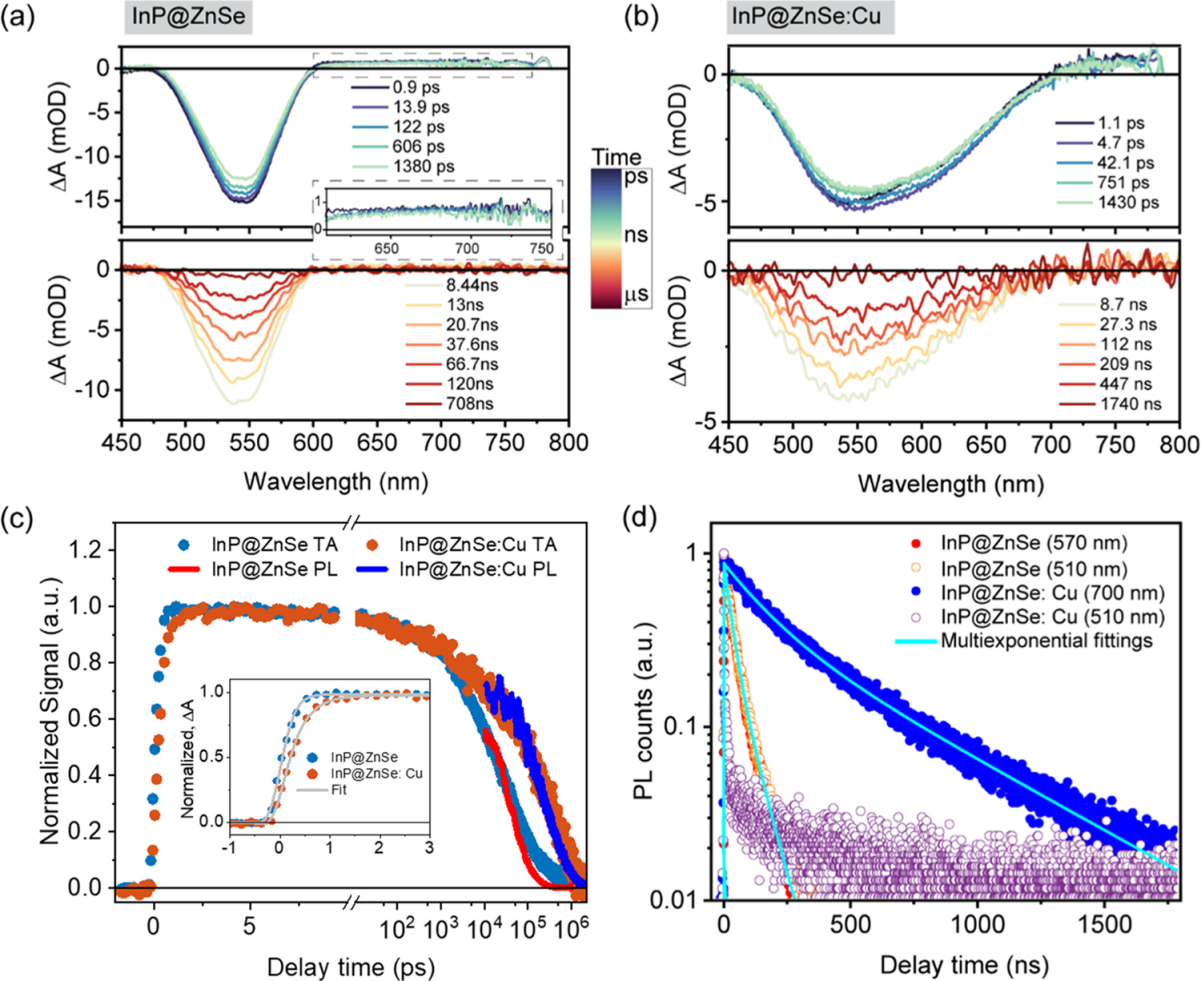
Impact of the Cu dopant on the intrinsic photophysical properties of InP QDs. (a, b) Transient absorption spectra of InP@ZnSe (a) and InP@ZnSe:Cu (b) at indicated delay times after 400 nm photoexcitation. Upper panel: delay within the ps–ns time range. Bottom panel: delay within the ns–μs time range. Inset of (a): zoomed-in spectral region (dashed box) between 600 and 750 nm. (c) XB decay of InP@ZnSe (dark cyan dots) and InP@ZnSe:Cu (orange dots) monitored at 540 nm. Inset: zoomed-in growth signal and their convoluted exponential fit (gray lines). The scaled PL decay curves of InP@ZnSe (red curve) and InP@ZnSe:Cu (blue) are also overlaid. (d) PL decay of InP@ZnSe and InP@ZnSe:Cu monitored at indicated wavelengths (colored solid or open dots), together with multiexponential fittings (cyan curves).

We first studied the impact of the Cu dopant on the intrinsic carrier dynamics of InP@ZnSe by combined transient absorption (TA) spectroscopy and time-resolved PL decay. Figure 2a,b shows the TA spectra of InP@ZnSe and InP@ZnSe:Cu QDs, respectively, at indicated delay times after photoexcitation at 400 nm (upper panel: ps–ns time range; lower panel: ns–μs time range). The TA spectra of InP@ZnSe QDs show a pronounced exciton bleach (XB) centered at ∼542 nm and a broad featureless photoinduced absorption (PA) region extending from 600 nm to longer wavelengths (Figure 2a, inset). These spectral features are similar to those observed in the TA spectra of bare InP QDs,^36^ with XB and PA assigned to the state-filling effect and the photoinduced absorption of 1S_e_ electrons, respectively. In comparison, the TA spectra of InP@ZnSe:Cu QDs show an additional bleach at 628 nm in addition to the abovementioned XB band. The position of this additional bleach agrees with the STE band in the static absorption spectrum (Figure 1a) and can be assigned to the state-filling effect of the 1S_e_ electron on the STE transition. Thus, this TA signal is named as the self-trapped exciton bleach, STEB. The identical decay kinetics of InP@ZnSe:Cu probed at both XB and STEB (Figure S2) further support this assignment.

To determine the trapping time of the photogenerated hole into the Cu dopant in InP@ZnSe:Cu, we compared the formation and decay kinetics of 1S_e_ electrons in both QDs probed at 540 nm. Under 400 nm above-bandgap excitation, the increase of 1S_e_ signals in QDs represents the cooling of higher energetic electrons from the 1P_e_ to the 1S_e_ state.^34,37,38^ As shown in Figure 2c inset, the rise time of InP@ZnSe:Cu (0.39 ± 0.02 ps) is noticeably slower than that of InP@ZnSe QDs (0.22 ± 0.02 ps). A similar increase in the electron cooling lifetime was also previously observed in Cu-doped CdSe QDs and has been attributed to ultrafast (≪390 fs) trapping of photogenerated holes from the valence band into the Cu state.^34^ Efficient cooling of hot conduction band electrons in undoped QDs has been attributed to the Auger type of process involving the valence band hole due to a strong electron–hole Coulombic interaction.^39^ However, the ultrafast trapping of holes to the Cu site on the femtosecond time scale removes this pathway and slows hot electron cooling in doped QDs. Furthermore, consistent with this ultrafast hole trapping, the band edge PL decay of the InP@ZnSe:Cu QDs (510 nm) was found to decay instantaneously within our instrument limits (Figure 2d). These results thus explain the absence of band edge emission in static PL measurements (Figure 1b) and confirm that photoexcitation in InP@ZnSe:Cu QDs leads to the formation of a “self-trapped exciton” on a subpicosecond time scale. We note that a recent study in Cu-doped InP/ZnSe/ZnSe_0.5_S_0.5_/ZnS core/shell QDs also shows a similar electron cooling time under the same excitation: 250 fs in undoped QD and 400 fs in Cu-doped QD.^40^ However, the hole trapping lifetime was assigned to be 1.8 ps,^40^ slower than the ultrafast hole trapping proposed here and in the literature.^34^ This difference may originate from different Cu dopant positions and core/shell structures.

Once formed, the self-trapped exciton in InP@ZnSe:Cu QDs is found to have a lifetime much longer than that of InP@ZnSe QDs. As shown in Figure 2c, the XB decay of InP@ZnSe:Cu is slower than that of InP@ZnSe, and the PL decay lifetime (Figure 2d) of the STE band (at 700 nm) of InP@ZnSe:Cu is also longer than that of the FE band (at 570 nm) of InP@ZnSe. Fitting of the PL decay by multiexponential functions reveals that the amplitude-weighted average lifetime constants of FE in InP@ZnSe and STE in InP@ZnSe:Cu are 48.3 ± 1.7 and 369.0 ± 4.3 ns, respectively. These long-lived self-trapped excitons in InP@ZnSe:Cu are in principle beneficial for driving sluggish chemical reactions and reasonably explain the superior performance of InP@ZnSe:Cu than that of the undoped QDs.^20,21,41^ However, as discussed below, additional complexities should be considered.

### Hole Transfer from QDs to Acceptors

**Figure 3:**
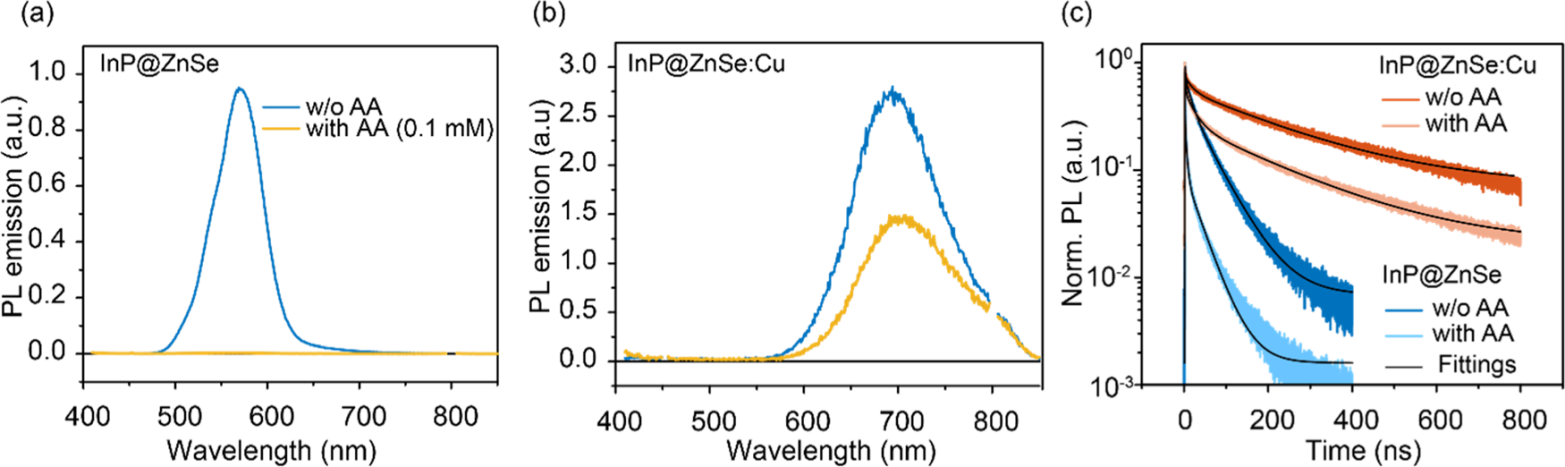
Impact of the Cu dopant on the hole transfer process of InP@ZnSe QDs. Steady-state PL emission changes of InP@ZnSe (a) and InP@ZnSe:Cu (b) QDs with the addition of AA as hole scavengers. (c) PL decay of InP@ZnSe and InP@ZnSe:Cu QDs with and without AA. Overlaid black lines are the multiexponential fittings.

To evaluate the impact of self-trapped excitons on the hole transfer (HT) process, we add ascorbic acid (AA) (a widely used hole acceptor in photocatalytic studies) into both QD solutions. Steady-state PL quenching and PL decay kinetics of the QDs are measured to characterize the hole transfer rates. The redox potential of AA ranges from 0 to +0.4 V vs NHE in the literature.^42−44^ Herein, we take the average value of +0.2 V for the hole extraction discussion below, as illustrated in Scheme 1. A methanol solution of AA was added into QD hexane solutions to obtain 0.1 mM AA in QD solutions. The detailed sample preparation is described in Section S1. As shown in Figure 3a,b, the addition of AA causes a complete quench of the FE PL of InP@ZnSe, but only ∼50% quench of the STE PL in InP@ZnSe:Cu QDs. Furthermore, PL decay measurements reveal that the addition of AA into these two QD solutions accelerates their PL decays (Figure 3c), consistent with hole extraction by AA. After the addition of AA, the amplitude-weighted lifetime constant of the fitted PL decay changes from 30.26 ± 0.12 to 1.51 ± 0.01 ns in InP@ZnSe QDs, while it decreases from 129.1 ± 0.2 to 70.1 ± 0.1 ns in InP@ZnSe:Cu. This corresponds to QD-to-AA HT rate constants (*k*_HT_) of (6.28 ± 0.02) × 10^8^ s^–1^ and (6.51 ± 0.03) × 10^6^ s^–1^ for InP@ZnSe and InP@ZnSe:Cu, respectively. Note that the shorter PL lifetimes of bare QDs in Figure 3c compared to those in Figure 2d may result from different solvents. A hexane and methanol mixture was used as the solvent for bare QDs in Figure 3 as a control to directly study the HT to AA, where methanol may also extract holes from QDs to accelerate the PL decay.^45^ Indeed, the QD-to-methanol *k*_HT_ is also faster in InP@ZnSe [(1.23 ± 0.0) × 10^7^ s^–1^] than in InP@ZnSe:Cu [(5.04 ± 0.03) × 10^6^ s^–1^]. As such, these results suggest orders of magnitude slower HT from InP@ZnSe:Cu QDs than that from InP@ZnSe QDs. Another commonly utilized hole scavenger, triethanolamine (TEOA), also leads to a faster quench of FE in InP@ZnSe than the STE in InP@ZnSe:Cu with quenching rates of 4.75 ± 0.06 and 1.08 ± 0.03 mM^–1^, respectively (Figure S3).

### Electron Transfer and Charge Recombination

**Figure 4:**
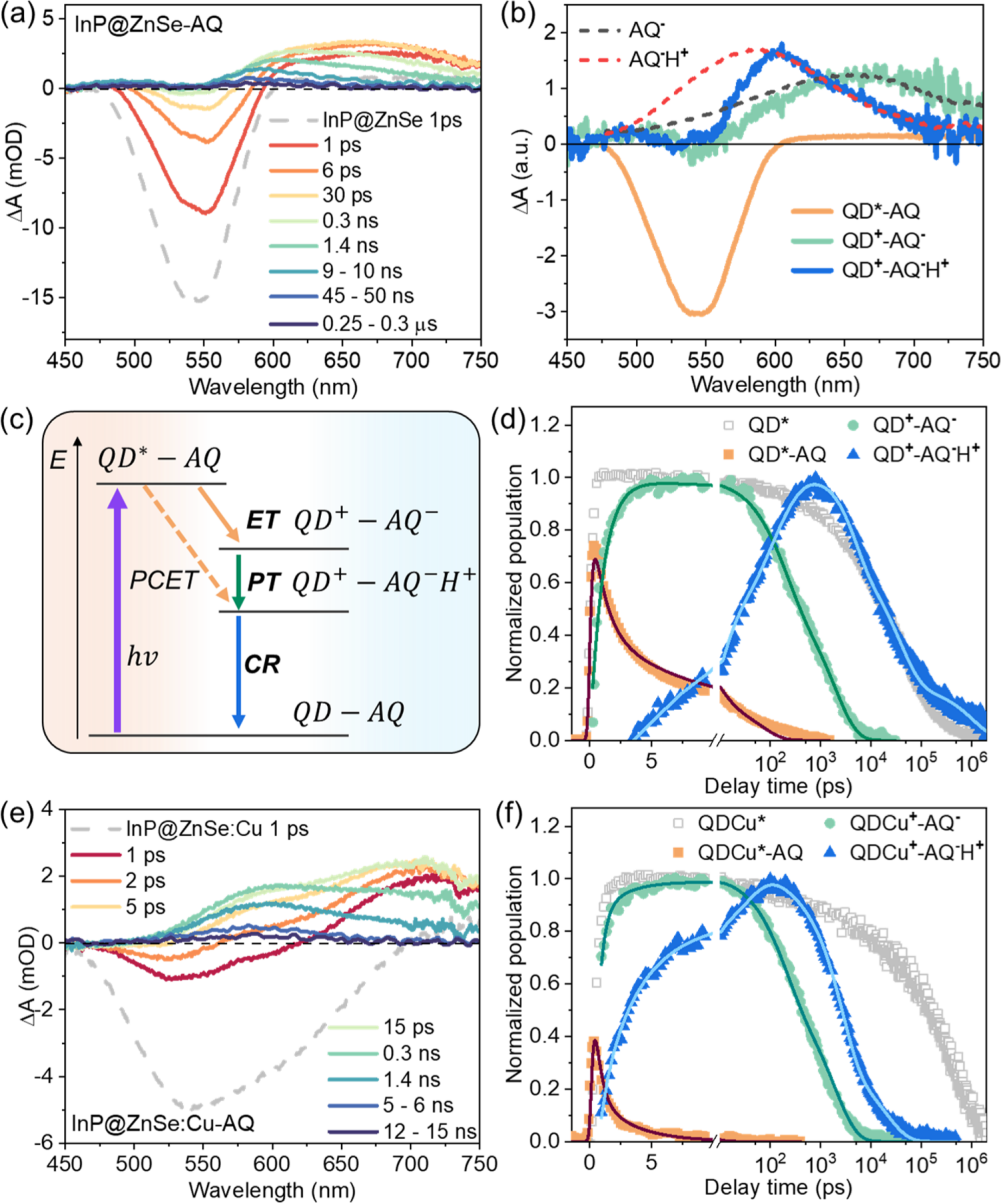
Impact of Cu dopant on electron transfer (ET) and charge recombination (CR) processes of InP@ZnSe QDs. (a, e) TA spectra of InP@ZnSe-AQ (a) and InP@ZnSe:Cu-AQ (e) at indicated delay times after photoexcitation at 400 nm. TA spectra of bare QDs without AQ at 1 ps are shown as gray dashed curves for comparison. (b) TA spectrum of the excited-state components in InP@ZnSe-AQ: excited QD [QD*-AQ, orange curve], charge-separated state before the blue shift or before protonation (QD^+^-AQ^–^, green curve), and charge-separated state after the blue shift or after protonation (QD^+^-AQ^–^H^+^, blue curve). Also shown are the reference spectra of AQ^–^ (black dashed curve) and AQ^–^H^+^ (red dashed curve). (c) Schematic of the excited-state dynamics in InP@ZnSe-AQ. (d, f) Normalized population kinetics of each excited-state component in InP@ZnSe-AQ (d) and InP@ZnSe:Cu-AQ (f) obtained from linear regression fitting: QD(Cu)*-AQ, orange solid squares; QD(Cu)^+^-AQ^–^, green dots; QD(Cu)^+^-AQ^–^H^+^, blue triangles. The colored solid lines are fittings to the population kinetics. Pure QD kinetics [QD(Cu)*, gray open squares] are compared to show the initial amplitude loss in the presence of AQ.

To evaluate the effects of Cu doping on the ET and the following charge recombination (CR) processes, we added the molecular electron acceptor anthraquinone-2,3-dicarboxylic acid (AQ) (Scheme 1) into QD solutions and used TA spectroscopy to measure the ET kinetics. As shown below, reduced AQ (AQ^–^) has characteristic optical signals that can be utilized to follow the charge separation and recombination processes. The redox potential of AQ is noted in Scheme 1b.^46^Figure S4 shows that the addition of AQ causes negligible changes to QD absorption. As shown in Figure 4a, after 400 nm excitation, the XB signal in the InP@ZnSe-AQ complex shows an initial amplitude loss within 1 ps, together with an instantaneously formed broad PA signal centered at 655 nm, which corresponds to the formation of AQ^–^.^46−49^ These spectral changes suggest an ultrafast ET from the InP@ZnSe QD to surface-adsorbed AQ molecules. From 1 to 30 ps, the XB decays with the growth of AQ^–^, further confirming the ET process and the formation of the charge-separated state (hole in QD and electron in AQ, QD^+^-AQ^–^). From 30 ps to 1.4 ns, the PA signal peak blue shifts from 655 to 600 nm, which then remains unchanged for the rest of the delay times (Figure S5). The PA peak shift resembles the spectral blue shift of semiquinone radical anions upon protonation to form the neutral semiquinone radicals.^50−53^ We note that in the QD-AQ complex samples, 5% methanol is introduced to facilitate the loading of AQ molecules onto the QD surface (Section S1). Upon ET to form the charge-separated state QD^+^-AQ^–^, methanol, as a proton source, may allow proton transfer (PT) to form the protonated charge-separated state QD^+^-AQ^–^H^+^. In addition, the carboxylic acid groups in AQ (Scheme 1b) may also be the proton source. In Figure 4b, the TA spectra of the charge-separated state before and after blue shifting are extracted and compared to the reference absorption spectra of the nonprotonated AQ radical anion (AQ^–^) and protonated AQ radical (AQ^–^H^+^), respectively. Details of the extracted spectra and reference spectra are provided in Sections S6.3 and S6.4. Figure 4b shows that, despite the Stark-effect-induced derivative signal near the XB position,^47^ the charge-separated state spectra agree well with the reference spectra, confirming the protonation as the reason for the observed spectral blue shift.

Figure 4c summarizes the photoinduced reactions in InP@ZnSe QD-AQ: the photoexcited QD-AQ complex (QD*-AQ) undergoes ultrafast ET to form the nonprotonated charge-separated state QD^+^-AQ^–^, followed by PT to form the protonated charge-separated state QD^+^-AQ^–^H^+^, which finally decays to the ground-state QD-AQ through CR. According to this model, the TA spectra in Figure 4a can be fit to the linear combination of these three excited-state components (QD*-AQ, QD^+^-AQ^–^, and QD^+^-AQ^–^H^+^) using a linear regression fitting method.^47,54,55^ The spectrum of each component is summarized in Figure 4b. The linear regression fitting is detailed in Section S6.5, and the TA spectra fitting results are shown in Figure S8. Figure 4d shows the population change of each component generated from the linear regression fitting. Note that the population maximum of QD*-AQ is not normalized to unity because of the observed initial amplitude loss compared to pure QD (Figure 4a, 1 ps TA spectrum; Figure 4d, QD* population). Details of quantifying the initial amplitude loss are shown in Section S6.6. Fitting the kinetics in Figure 4d to the model in Figure 4c (Section S7) reveals averaged ET and CR rate constants of 0.57 ± 0.03 ps^–1^ and 0.21 ± 0.01 ns^–1^, respectively. Interestingly, it is found necessary to involve the concerted proton-coupled ET (PCET) in the kinetic fitting model, as the slow PT-induced QD^+^-AQ^–^ decay cannot explain the fast growth part of the QD^+-^AQ^–^H^+^ kinetics (Figure 4d). Another possibility for this fast growth of QD^+^-AQ^–^H^+^ is partially protonated QD-AQ (QD-AQH^+^) in the ground state, which directly forms QD^+^-AQ^–^H^+^ after ET. As shown in Section S6.4, the potential PCET or ground-state protonation may be better studied using CdS QDs whose XB signal (<450 nm) will interfere less with the AQ acceptor signals. However, more detailed studies on PCET or proton transfer are beyond the scope of this work.

The TA spectra of InP@ZnSe:Cu-AQ (Figure 4e) show the same spectral evolution observed in InP@ZnSe-AQ: an initial amplitude loss of the STEB signal within 1 ps, the concomitant STEB decay and PA (>550 nm) growth due to ET at <15 ps, and the blue shift of the PA peak occurring from 15 ps to 1.4 ns due to PT. Thus, the same kinetics model (Figure 4c) and spectral analysis are applied to the TA data of InP@ZnSe:Cu-AQ. The TA spectra of each excited-state component are shown in Figure S6b, including the photoexcited InP@ZnSe:Cu-AQ complex (QDCu*-AQ), the nonprotonated charge-separated state (QDCu^+^-AQ^–^), and the protonated charge-separated state (QDCu^+^-AQ^–^H^+^). The kinetics of each component obtained from linear regression fitting is shown in Figure 4f. It is worth noting that the decay part of the QDCu^+^-AQ^–^ and QD^+^-AQ^–^ kinetics are the same (Figure S10), showing no dependence on Cu doping or the remaining hole state in the QD. These results are consistent with the proposed protonation step, which mainly depends on the acceptor’s p*K*_a_ and the local pH.^51,52^ The kinetics of InP@ZnSe:Cu-AQ and InP@ZnSe-AQ are then globally fitted using the shared PT rate constants. Details can be found in Section S7. The fitting reveals averaged ET and CR rate constants of 1.76 ± 0.08 ps^–1^ and 22.1 ± 2.6 ns^–1^, respectively, in InP@ZnSe:Cu-AQ.

Another common electron acceptor, methyl viologen dichloride (MV^2+^),^56,57^ is also utilized to study the Cu dopant effect on ET and CR. The TA spectra and kinetics are summarized in Figure S12. The ET (CR) rate constants in InP@ZnSe-MV^2+^ and InP@ZnSe:Cu-MV^2+^ are 3.12 ± 0.07 ps^–1^ (2.72 ± 0.32 ns^–1^) and 2.72 ± 0.30 ps^–1^ (4.08 ± 0.75 ns^–1^), respectively. Figure S13 compares the reduced electron acceptor kinetics with and without the Cu dopant. While the ET rate constants show no clear dependence on Cu doping, the CR rate constants increase with Cu doping for both electron acceptors.

## Discussion

The measured exciton lifetimes and rate constants of hole transfer, electron transfer, and charge recombination in InP@ZnSe and InP@ZnSe:Cu QDs are listed in Table 1. Note that the number of acceptors per QD in the samples with and without Cu doping should be similar, as expected from the same QD surface condition, the similar QD diameters, and the same sample preparation procedure (Section S6.1). Thus, the measured rate constants can be directly compared to study the Cu dopant effect. The corresponding charge transfer efficiencies are summarized in Table S3. As mentioned above, the ET rate constants are independent of Cu doping for the studied electron acceptors. These results are consistent with the strong confinement nature of electrons in InP QDs with an exciton Bohr radius of ∼10 nm.^58,59^ As such, any possible change of Coulombic interaction due to the presence of Cu is not expected to significantly affect both the spatial and energy distributions of their electron wave function. Accordingly, Table S3 shows that the electron transfer efficiencies are near unity for all of the studied QDs and electron acceptors. On the other hand, it is clear from Table 1 that Cu doping slows the hole transfer and accelerates the charge recombination between the hole in the QD and electron in the acceptor, both of which are not favored for optimized charge transfer in photocatalytic or photovoltaic applications. As shown in Table S3, although the extended exciton lifetime in doped QDs may tolerate the slower hole transfer and show higher hole transfer efficiency in the absence of electron transfer, the slower hole transfer may be outcompeted by the faster charge recombination in doped QDs in the charge-separated state and lead to a much lower charge separation efficiency. These results may account for the deteriorated photocatalytic performance of Cu-doped nanocrystals reported previously.^22^ It should also be noted that tuning the Cu dopant amount in the current InP@ZnSe:Cu QDs does not change the charge transfer kinetics (Section S9.3). Below, we discuss the possibly involved mechanisms.

**Table 1:** Exciton Lifetime and Charge Transfer Rate Constants in InP@ZnSe and InP@ZnSe:Cu QDs

		InP@ZnSe	InP@ZnSe:Cu
exciton lifetime/ns		48.3 ± 1.7	369.0 ± 4.3
hole transfer/μs^–1^	AA	628 ± 2	6.51 ± 0.03
	methanol	12.3 ± 0.1	5.04 ± 0.03
	TEOA^a^	4.75 ± 0.06	1.08 ± 0.03
electron transfer/ps^–1^	AQ	0.57 ± 0.03	1.76 ± 0.08
	MV^2+^	3.12 ± 0.07	2.72 ± 0.30
charge recomb./ns^–1^	AQ^–^H^+^	0.21 ± 0.01	22.1 ± 2.6
	MV^+•^	2.72 ± 0.32	4.08 ± 0.75

a The hole transfer to TEOA is characterized by the Stern–Volmer quenching constant with the unit mM^–1^.

Charge transfer between QDs and surface molecules can be interpreted by the nonadiabatic Marcus theory^60^
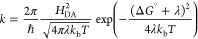
where λ is the reorganization energy of the charge transfer, Δ*G*^0^ is the associated free energy change, and *k*_b_ is the Boltzmann constant. *H*_DA_ is the coupling between the donor and acceptor states, which is proportional to the hole densities at the surface in the case of hole extraction and charge recombination, with the surface densities of free exciton hole and trapped hole designated to be ρ_h,free_ and ρ_h,trap_, respectively.

Hole trapping into Cu sites could alter the interfacial hole transfer by two ways: (a) change the hole wave function density at the surface from ρ_h,free_ to ρ_h,trap_, i.e., altering the wave function overlapping, *H*_DA_, and (b) change the energies of holes in the systems, with the energy of trapped holes ∼0.38 eV above the 1S_h_ level of InP@ZnSe, thus affecting the reaction driving force, Δ*G*^0^.

Herein, hole localization into Cu states results in a slower HT with hole scavengers but faster recombination between reduced electron acceptors and holes in NCs. Notably, these processes involve the same localized hole state but with different surface molecular species. As such, the hole wave function localization into Cu is less likely the major factor affecting the interfacial charge transfer because it would result in similar impact on the *H*_DA_ term for both HT and charge recombination, inconsistent with the experimental observation. Thus, the change in the hole energetic level is likely the dominating factor for the observed charge transfer changes. In QD-molecular assembles, HT is normally considered within the normal region of Marcus theory due to its smaller driving force than the reorganization energy; thus, a smaller driving force should result in a slower transfer rate. In comparison, charge recombination between reduced molecules and the hole in NCs has been suggested to occur in the inverted region of Marcus theory, where a smaller driving force should cause a faster recombination dynamics, as recently observed in a series study of CdS QDs and CdSe nanoplatelets.^61^ Herein, for charge recombination with AQ^–^H^+^, the driving force is approximately −0.78 to −0.90 eV (Section S10.3). The reorganization energy is estimated on the order of 0.32 eV, including contributions from inner-sphere reorganization of the acceptor (estimated to be ∼0.21 eV by density functional theory calculations in Section S10 and Table S4, same below) and solvent reorganization (∼0.11 eV) with negligible contribution from the QD.^46,62^ The much larger |Δ*G*^0^| (0.78–0.90 eV) than λ (∼0.32 eV) agrees with the Marcus inverted region of the charge recombination. On the other hand, the Δ*G*^0^ and λ for the hole extraction reactions with AA are ∼−0.39 eV (Scheme 1b and Table S7) and ∼0.49 eV (Table S4), respectively, indicating the normal regions, in good agreement with the observed experimental results. Similar analyses using the Marcus theory explain the slower HT to methanol and the faster charge recombination with MV^+•^ in Cu-doped QDs, as summarized in Figure S16. We note that an increase of the reorganization energy of the Cu^+^ dopant^40^ in the QDs negligibly affects the Cu doping impact on the charge transfer. In addition, Table 1 shows that for Cu-doped QDs, HT to AA and methanol is decelerated by 96.5 ± 0.5 and 2.44 ± 0.02 times, respectively, and charge recombination with AQ^–^H^+^ and MV^+•^ is accelerated by 105 ± 13 and 1.5 ± 0.3 times, respectively. These different Cu doping effects on different acceptors can also be explained within the Marcus theory. More detailed discussions on the driving force, reorganization energy, and dopant density are given in Section S11.

Furthermore, hole localization into the Cu state is shown to prolong the lifetime of the self-trapped exciton, probably due to the dramatically reduced electron–hole wave function overlapping, especially when the Cu^+^ dopant is farther from the QD center (Table S6). However, this does not necessarily mean a less wave function overlapping between the hole and surface molecules. Recent density functional theory studies on CdSe and ZnS have shown that Cu doping can generate a hole localization with higher densities at the surface.^19,63,64^ This is particularly true for core–shell NCs, where the intrinsic hole is mostly localized within the core and its wave function at the surface decreases exponentially upon increasing the shell thickness. In Section S10, we established a modified effective mass model, which considers the presence of Cu^+^ as a local potential well. The model predicts the energy level of Cu^+^ ∼ 0.36 eV above the intrinsic 1S_h_ level, in good agreement with experimental observations (Figure 1b). More importantly, it estimates that the hole wave function densities at the surface could be enhanced by a factor of 10 when placing the Cu^+^ at ∼0.2 nm inside the shell, with negligible changes in electron wave functions (Table S6). The enhanced hole densities at the surface indicate that tuning the position of dopants could promote the coupling between the photogenerated hole and surface acceptors in doped NCs as compared to those in intrinsic QDs, which may ultimately compensate for the side-effects due to the loss of the driving force. On the other hand, increasing the number of dopants at the same radial distance to the core does not affect the surface hole wave function density, explaining the same charge transfer kinetics observed in QDs with different Cu:In ratios (Figure S14). Thus, future work that targets fine-tuning of the dopant radial location should be promising to achieve balanced driving force loss and increased coupling between the surface molecules and colloidal nanocrystals, which is beneficial for a wide range of applications.

## Conclusions

In conclusion, the present study used the green, nontoxic InP@ZnSe QDs as a platform to systematically study the impact of the Cu dopant on the photophysical and interfacial charge transfer dynamics of InP QDs. Our results show that the inclusion of the Cu dopant into InP@ZnSe can create self-trapped excitons, which extends the exciton lifetime from 48.3 ± 1.7 to 369 ± 4.3 ns. However, hole transfer from this self-trapped exciton state is retarded compared to that from the free exciton state. Furthermore, although ET between QDs and electron acceptors is independent of Cu doping, the subsequent charge recombination between the reduced surface molecules and the holes in the QDs becomes significantly faster in InP@ZnSe:Cu quantum dots. These results can be rationalized by the higher hole energy level in InP@ZnSe:Cu, reducing the driving force for hole extraction and charge recombination. Overall, these results illustrate the impact of dopants on individual charge transfer steps and can guide further rational design of doped nanocrystals for optimized optoelectronic applications.

## Supporting Information Available

Sample preparation, experimental methods, fitting of TA spectra and kinetics, and calculation of QD energy levels and reorganization energies (PDF)
