# Supplementary material for: Doping of Colloidal Nanocrystals for Optimizing Interfacial Charge Transfer: A Double-Edged Sword
Source: J Am Chem Soc. 2024 Aug 27;146(36):24925–34. doi: 10.1021/jacs.4c06110 (PMC11403596; doi:10.1021/jacs.4c06110)
Supplement: Supplementary file 1 — ja4c06110_si_001.pdf [file ja4c06110_si_001.pdf]

Supporting Information for

Doping of colloidal nanocrystals for optimizing interfacial charge transfer: a double-edged sword

Sheng He[†], Anji Ni[†], Sara T. Gebre[†], Rui Hang[†], James R. McBride[¶], Alexey L. Kaledin[†#*], Wenxing Yang[†*], Tianquan Lian[†*]

† Department of Chemistry, Emory University, 1515 Dickey Drive Northeast, Atlanta, Georgia 30322, USA

¶ Department of Chemistry, The Vanderbilt Institute of Nanoscale Science and Engineering, Vanderbilt University, Nashville TN, 37235, USA

The Cherry L. Emerson Center for Scientific Computation, Emory University, 1515 Dickey Drive Northeast, Atlanta, Georgia 30322, USA

Contents

S1. Sample preparation....3
S2. Experiment setup....4
S3. Transmission electron microscopy (TEM) measurements....5
S4. TA kinetics of InP@ZnSe:Cu....5
S5. PL quench by TEOA....6
S6. TA spectra analysis of QD(Cu)-AQ samples....6
S6.1 UV-vis absorption spectrum of QD-AQ and QD-MV^{2+} complexes and discussion on the number of surface acceptors....6
S6.2 Long delay time TA spectra of QD-AQ samples....7
S6.3 TA spectra of the pure QD component and the charge separated states before and after blue shift....7
S6.4 Reference spectrum of AQ^- and AQ^-H^+....9
S6.5 Linear regression fitting of the TA spectra of InP@ZnSe-AQ and InP@ZnSe:Cu-AQ....11
S6.6 Scaling the QD*-AQ population according to the measured initial amplitude loss....12
S6.7 Compare the QD^+-AQ^- and $QDCu^+$-AQ^- kinetics....13
S7. TA kinetics fitting in QD(Cu)-AQ samples....13
S8. TA study of ET in QD-MV^{2+} complexes....17
S9. Discussion on charge transfer rate constants and efficiencies....20
S9.1 Compare electron transfer and charge recombination kinetics....20
S9.2 Charge transfer efficiencies....20
S9.3 No dependence of electron transfer and charge recombination on Cu: In ratio....21
S10. Calculations of energy levels and surface charge densities....22
S10.1 Calculations of reorganization energies....22
S10.2 Calculations of electron and hole energy levels and surface charge densities....23
S10.3 Driving force of charge recombination in InP@ZnSe-AQ....26
S11. Effect of driving force, reorganization energy, and dopant density on rate constants....27
S11.1 Effect of driving force and reorganization energy....27
S11.2 Effect of dopant density....31

S1. Sample preparation.

Quantum dot (QD) synthesis

In our trial synthesis, we found that the shelling of ZnS by the same methods produce thinner shells. Thus, in the current systems, we use ZnSe shell instead. The InP@ZnSe QDs were synthesized according to literature with modifications.[1]

Typically, 0.4 mmol indium acetate [In(Ac)$_3$], 1.4 mmol myristic acid and 5 mL 1-octadecene (ODE) were loaded into a 50 mL three-neck flask and degassed under vacuum at 110 ℃ for 1 hour with rigorous stirring. The flask was then refilled with Argon and the temperature was raised to 210 ℃. The growth of the InP core was induced by injecting the P precursor (0.2 mmol tris(trimethylsilyl)phosphine, (TMS)$_3$P, and 2.4 mmol octylamine in 1.5 mL ODE, prepared in glovebox) into the flask. The reaction proceeded for 5 minutes and was quenched by cooling the flask to 150℃. To grow a ZnSe shell on the as-synthesized InP core QDs, successive ion layer adsorption and reaction (SILAR) was applied. For the first run of SILAR, 1.2 mL of the Zn precursor (0.1 M zinc stearate in ODE, preheated and degassed at 100℃ for 1 hour) was added at 150℃. After 10 minutes, the same amount of the Se precursor (1.2 mL, 0.1 M Se in ODE, degassed via Argon bubbling) was added and the temperature was increased to 220 ℃ for 30 minutes to allow the growth of the ZnSe shell. Then the temperature was decreased to 150℃ to start the next SILAR process. For the second and third run of SILAR, 1.65 mL and 2.1 mL of each precursor were added, and the same thermal cycling was repeated. The reaction was finally stopped by cooling the flask to the room temperature via water bath.

To purify the synthesized InP@ZnSe QDs, the reaction solution was first centrifuged at 5500 rpm for 10 minutes to remove unreacted salts. The supernatant was then mixed with acetone (twice in volume) and centrifuged at 5500 rpm for 10 minutes to precipitate the QDs, which was then redispersed in hexane. To further purify the QDs, ethanol was added into the hexane solution to obtain a turbid liquid, which was centrifuged at 5500 rpm for 10 minutes. The precipitated QDs were dispersed in hexane for future measurement, and some possible insoluble solids can be removed by further centrifugation.

For the synthesis of the InP@ZnSe: Cu QDs, copper doping was conducted after the first SILAR run when synthesizing the InP@ZnSe QDs. Specifically, after staying at 220 ℃ for 30 minutes for the first growth of the ZnSe shell, the reaction solution was cooled down to 130℃, and 1.2 mL of the Cu precursor (0.1 M copper stearate in ODE, degassed via Argon bubbling) was injected. Then the temperature was increased to 210℃ for 1 hour to allow for the copper doping, after which the temperature was decreased to 150 ℃ to continue the second and third SILAR process. The purification of the InP@ZnSe: Cu QDs was the same as that of the InP@ZnSe QDs.

QD-acceptor complexes

For the hole transfer study, 150 μL of the above QD solutions was diluted into a 2.5 mL hexane solution, to which 15 μL 16.7 mM ascorbic acid (AA) in methanol was added, resulting in the 0.1 mM final concentration of AA in the QD solution. For the QD samples without AA, the

same dilution was conducted and same amount of methanol was added as control to study HT to AA.

For the electron transfer study, 20 μL of 250 μM anthraquinone-2,3-dicarboxylic acid (AQ) methanol solution was added into 400 μL QDs solutions to form the QD-AQ complex samples. For the control sample, 20 μL methanol was added to the QDs solutions. In the case of methyl viologen (MV^{2+}), 7.5 μL 50 mM MV^{2+} methanol solution was added into QDs solutions, followed by sonication for 1 minute. The mixed solution was than filtered through a 200 μm syringe filter to remove any insoluble solids.

S2. Experiment setup.

Time-resolved photoluminescence (TRPL) setup

The TRPL setup is built with a mode-locked Ti: Sapphire laser (Tsunami oscillator, Spectra Physics). The laser output is selected as 800 nm with a repetition rate of 80 MHz. The repetition rate is modified by an E-O modulator (ConOptics, Model 350-160) to match the PL lifetimes of different samples. The laser pulse is then directed into a BBO crystal to generate the 400 nm laser through the second harmonic generation effect. The 400 nm laser with a modified repetition rate is used as the excitation pulse to generate the PL. The emitted PL photons from the sample are collected into a monochromator (Acton Series, Princeton Instruments), which can select the wavelength of the output PL photons. After the monochromator, the selected photons were collected by a microchannel-plate-photomultiplier tube (Hamamatsu R3809U-51), which is connected to a time-correlated single photon counting (TCSPC) board (Becker & Hickel SPC 600).

Transient absorption (TA) setup

Femtosecond TA spectroscopy. TA measurements were performed with a Helios spectrometer (Ultrafast Systems LLC). The 800 nm fundamental light was generated by a Ti: Sapphire laser (Coherent Legend, 150 fs, 3 mJ/pulse, and 1 kHz repetition rate) and split into two parts. One part of 800 nm beam was attenuated and focused on a sapphire window for the generation of the white light continuum. The reference and probe beams were focused into a fiber optics-coupled multichannel spectrometer with complementary metal-oxide-semiconductor (CMOS) sensors and detected at a frequency of 1 kHz. Another part of the 800 nm beam is focused into a frequency doubling BBO crystal to generate the 400 nm pump excitation. For other excitation wavelengths, the 800 nm was focused into an optical parametric amplifier (Light conversion, TOPAS) to generate desired wavelengths. The pump beam was chopped at 500 Hz. The delay time between the pump and probe pulse was controlled by a motorized delay stage. The change in the transmitted probe pulses for the pumped and unpumped samples was calculated and converted to the absorption difference. The samples were dispersed in a 1 mm cuvette and stirred vigorously during the measurement. The diameter of the pump beams was estimated, using the razor blade method, to be ~330 μm for the 400 nm pump.

Nanosecond TA spectroscopy. Nanosecond TA was performed with the EOS spectrometer (Ultrafast Systems LLC). The pump beam at 400 nm was generated in the same way as femtosecond TA experiments. The white light continuum (380−1700 nm, 0.5 ns pulse width, 20 kHz repetition rate) used here was generated by focusing an Nd:YAG laser into a photonic crystal

fiber. The delay time between the pump and probe beam was controlled by a digital delay generator (CNT-90, Pendulum Instruments). The probe and reference beams were detected with the same multichannel spectrometers used in femtosecond TA experiments. The IRF of this system was measured to be ~280 ps.

S3. Transmission electron microscopy (TEM) measurements.

TEM images were collected using a Tecnai Osiris 200 kV TEM. TEM samples were prepared by drop-casting dilute solutions of the particles on to carbon coated nickel TEM grids (Electron Microscopy Sciences, LC300-NI-FCF). Typical TEM images of the InP@ZnSe and InP@ZnSe:Cu QDs are shown in Figure S1.

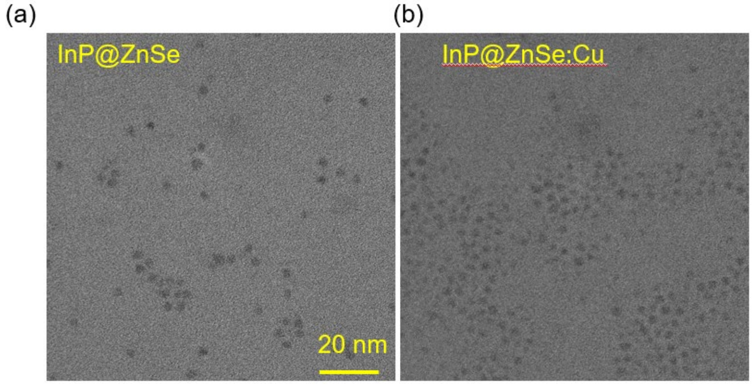

Figure S1. TEM of InP/ZnSe and InP/ZnSe:Cu studied in the present study.

S4. TA kinetics of InP@ZnSe:Cu.

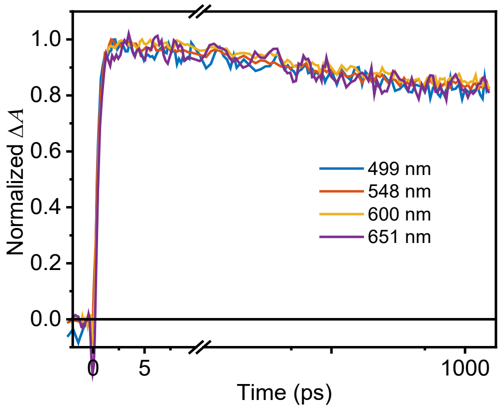

Figure S2. STEB decay dynamics of InP/ZnSe:Cu detected at different wavelengths when photoexcited at 400 nm.

S5. PL quench by TEOA.

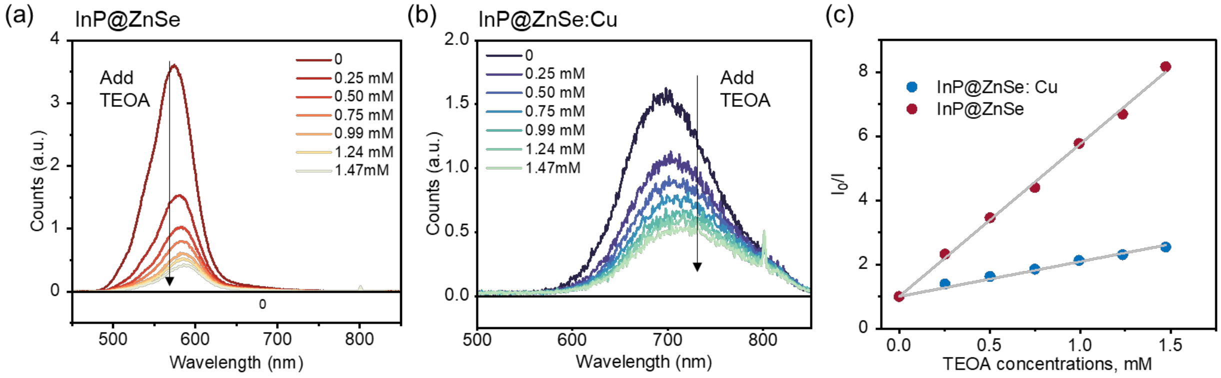

Figure S3. PL changes of InP@ZnSe (a) and InP@ZnSe:Cu (b) upon the gradual addition of TEOA into the solution. (c) the ratio between the initial PL intensity (I_0) and the PL intensity with TEOA added (I) as a function of TEOA concentration. The gray lines are linear fit to equation $I_0/I=1+k*C_{TEOA}$, where k defines the quench rate, mM^{-1}.[2]

S6. TA spectra analysis of QD(Cu)-AQ samples.

S6.1 UV-vis absorption spectrum of QD-AQ and QD-MV^{2+} complexes and discussion on the number of surface acceptors.

As shown in Figure S4, the addition of electron acceptors (AQ and MV^{2+}) into the QD solutions causes negligible changes to the absorption features of the QDs.

Although the acceptors' absorption is not available in wavelength range in Figure S4, their concentration for the sample with and without Cu should be the same as expected from the same sample preparation methods described in section S1.

It should be noted that in the synthesis of Cu-doped QDs, the Cu precursor (0.1 M copper stearate in ODE) is similar as the Zn precursor (0.1 M zinc stearate in ODE), so the doped QDs are believed to show the same surface conditions as the undoped QDs. As a result, in the presence of an excess of acceptors, which is indicated by the ultrafast electron transfer in the ps scale and the complete PL quench in InP@ZnSe QD by AA, the average number of acceptors on each QD surface can be treated as proportional to the QD surface area. From the measured diameters, the ratio of the two QDs' surface area is $S_{QD}:S_{QD,Cu} = 1.16$, close to 1. Although this result indicates 16% more acceptors on each undoped QD compared to the doped QD, as will be shown in Table 1, it cannot explain the faster hole transfer in the undoped QDs or affect the conclusion on electron transfer. In addition, the charge recombination kinetics is independent on the surface acceptor numbers because charge recombination only involves the reduced acceptor.

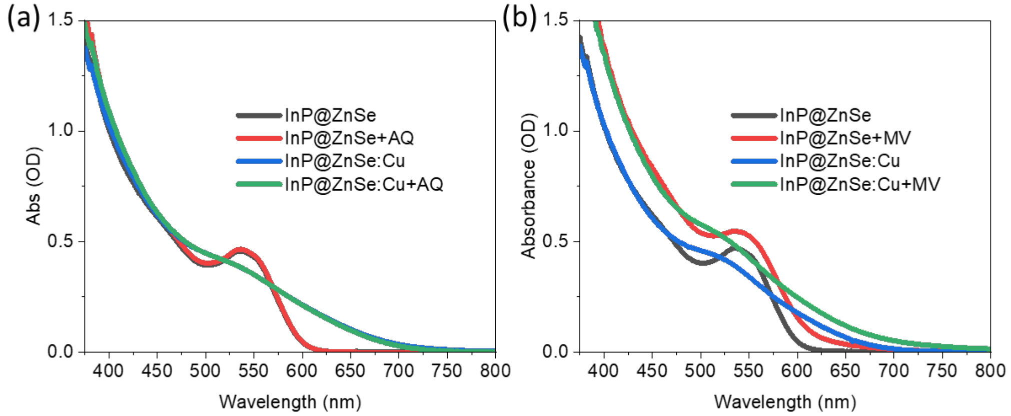

Figure S4. Absorption spectrum of the QD-AQ (a) and QD-MV^{2+} (b) complexes. The additional absorption after adding MV^{2+} is probably due to Relay scattering in the sample.

S6.2 Long delay time TA spectra of QD-AQ samples.

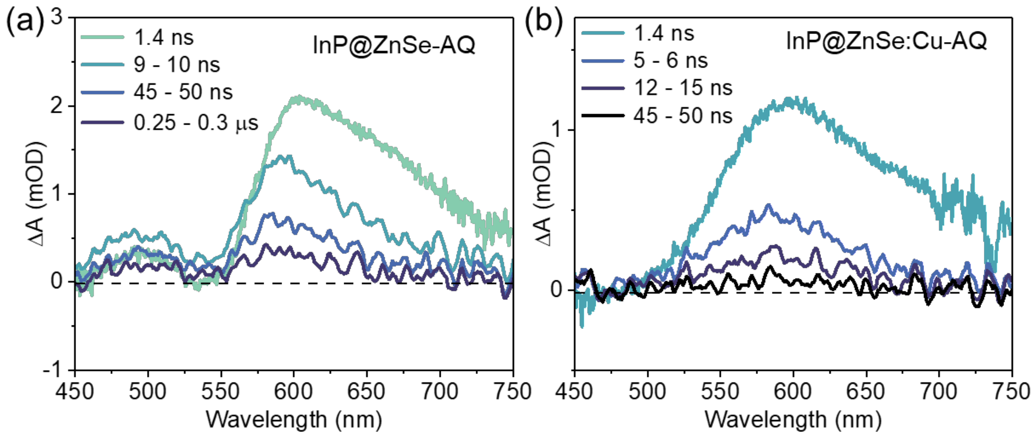

Figure S5. Long delay time TA spectra of InP@ZnSe-AQ (a) and InP@ZnSe:Cu-AQ (b). The spectral shape remains the same when the amplitude decreases after 10 or 6 ns.

S6.3 TA spectra of the pure QD component and the charge separated states before and after blue shift.

As discussed in the main text, the TA spectra change of the InP@ZnSe:Cu-AQ sample involves three components: the XB of the QD, the charge separated state signal before and after blue shift. To be consistent with the main text and later discussion, the three components are named as the following: "QD*-AQ" for the XB of the QD, "QD$^+$-AQ$^-$" for the charge separated state signal before blue shift, and "QD$^+$-AQ$^-$H$^+$" for the charge separated state signal after blue shift.

As shown in Figure S4, the addition of AQ does not change the UV-vis absorption spectrum of the InP@ZnSe QD. As a result, in InP@ZnSe-AQ, the QD*-AQ spectrum can be taken as the TA spectrum of bare InP@ZnSe QDs at 2 ps, as shown in Figure 4b.

The charge separated state signal after blue shift, or the QD^+-AQ^-H^+ spectrum, is taken as the TA spectrum of InP@ZnSe-AQ at long delay time, 25 ns, when the spectral shift finishes and only QD^+-AQ^-H^+ survives. Note that the QD^+-AQ^-H^+ spectrum shows a derivative feature around the XB peak position, 540 nm. This derivative feature is attributed to the Stark effect in the charge separated state.[3]

The extraction of the charge separated state signal before blue shift, or the QD^+-AQ^- spectrum, is shown in Figure S6a. It is observed that at very early delay times, from 1 ps to 3 ps, the TA spectra change only contains the decay of XB and the growth of PA centered at 650 nm, without the growth of PA centered at 600 nm or spectral blue shift. Thus, the difference between the 1 ps spectrum and 3 ps spectrum is a sum of the negative XB (QD*-AQ) signal and the QD^+-AQ^- signal. Further subtracting the XB contribution, which is represented by the flipped and scaled 2 ps spectrum of bare QD in Figure S6a, in the (3 ps – 1 ps) difference spectrum results in the QD^+-AQ^- spectrum. The extracted spectrum of QD*-AQ, QD^+-AQ^-, and QD^+-AQ^-H^+ are summarized in Figure 4b.

In InP@ZnSe:Cu-AQ, similar as the undoped case, the QDCu*-AQ spectrum is taken as the 2 ps TA spectrum of bare InP@ZnSe:Cu QD, and the $QDCu^+$-AQ^-H^+ spectrum is taken as the TA spectrum of InP@ZnSe:Cu-AQ averaged over long delay times (from 10 ns to 20 ns), as shown in Figure S6b. In this QD^+-AQ^-H^+ spectrum, negligible derivative features were observed, probably due to the broad feature of STEB and thus the less sharp Stark effect induced spectral features.

To obtain the $QDCu^+$-AQ^- spectrum, we notice that from 60 ps to 300 ps, the TA spectra only show decay above 600 nm (Figure S6c). This part of decay is attributed to the protonation of $QDCu^+$-AQ^-, and the difference between the 60 ps spectrum and the 300 ps spectrum is taken as the $QDCu^+$-AQ^- spectrum. The spectrum of QDCu*-AQ, $QDCu^+$-AQ^-, and $QDCu^+$-AQ^-H^+ in InP@ZnSe:Cu-AQ are summarized in Figure S6b. When compared to the reference spectrum of AQ^- and AQ^-H^+ (Figure S6b), the $QDCu^+$-AQ^-H^+ spectrum agree well with the AQ^-H^+ spectrum, and the $QDCu^+$-AQ^- spectrum agree with the AQ^- spectrum at long wavelength (>700 nm). The discrepancy at < 700 nm is attributed to the Stark effect in the charge separated state, which causes derivative features based on the broad STEB signal.

To further verify the $QDCu^+$-AQ^- and $QDCu^+$-AQ^-H^+ spectrum, we applied the Multivariate Curve Resolution (MCR) method to the time- and wavelength-dependent TA data to independently extract the spectral components.[4] In the MCR analysis, the TA data are fit to three spectral components with their own time evolution profiles. One of the three spectra is fixed as the 2 ps TA spectrum of bare InP@ZnSe:Cu QD, and the other two are generated by the MCR analysis. The MCR generated spectra are shown in Figure S6d, which are similar to those spectra obtained from the above spectra analysis in Figure S6b and Figure S6c, validating the obtained spectra in Figure S6b as the TA spectral components in InP@ZnSe:Cu-AQ.

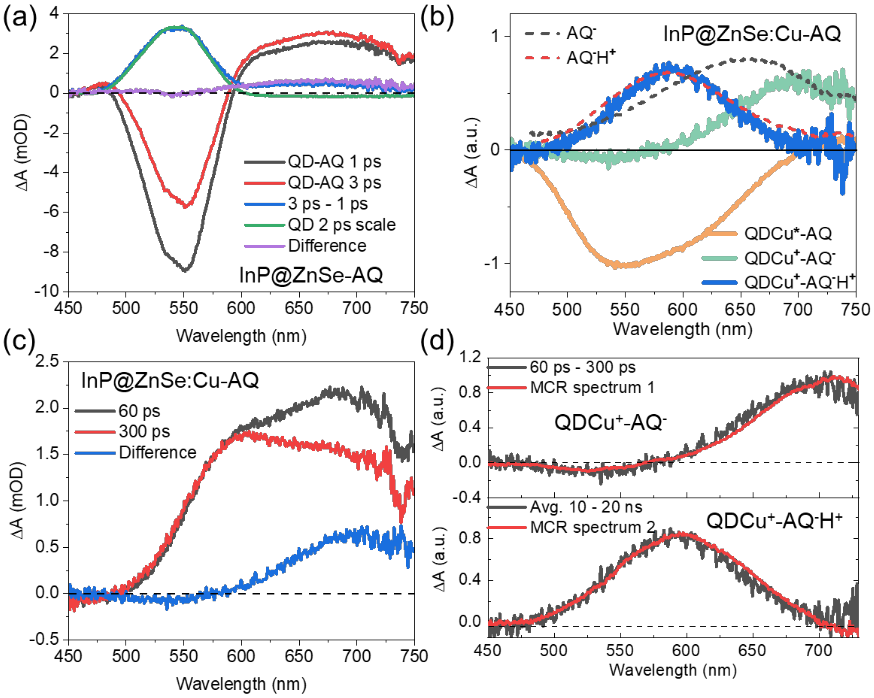

Figure S6. (a) Spectra used to obtain the QD^{+}-AQ^{-} spectrum in in InP@ZnSe-AQ. (b) TA spectral components of pure STEB [QDCu*-AQ, orange curve], charge separated state before blue shift or before protonation ($QDCu^{+}$-AQ^{-}, green curve), and charge separated state after blue shift or after protonation ($QDCu^{+}$-$AQ^{-}H^{+}$, blue curve) in InP@ZnSe:Cu-AQ. Also shown are the reference spectrum of AQ^{-} (black dashed curve) and $AQ^{-}H^{+}$ (red dashed curve). (c) Spectra used to obtain the $QDCu^{+}$-AQ^{-} spectrum in InP@ZnSe:Cu-AQ. (d) Upper panel: comparison between the MCR generated spectrum component NO. 1 (red) and the $QDCu^{+}$-AQ^{-} spectrum obtained from the difference between 60 ps and 300 ps (black); lower panel: comparison between the MCR generated spectrum component NO. 2 (red) and the $QDCu^{+}$-$AQ^{-}H^{+}$ spectrum obtained by averaging the 10 ns to 20 ns spectrum (black).

S6.4 Reference spectrum of AQ^{-} and $AQ^{-}H^{+}$.

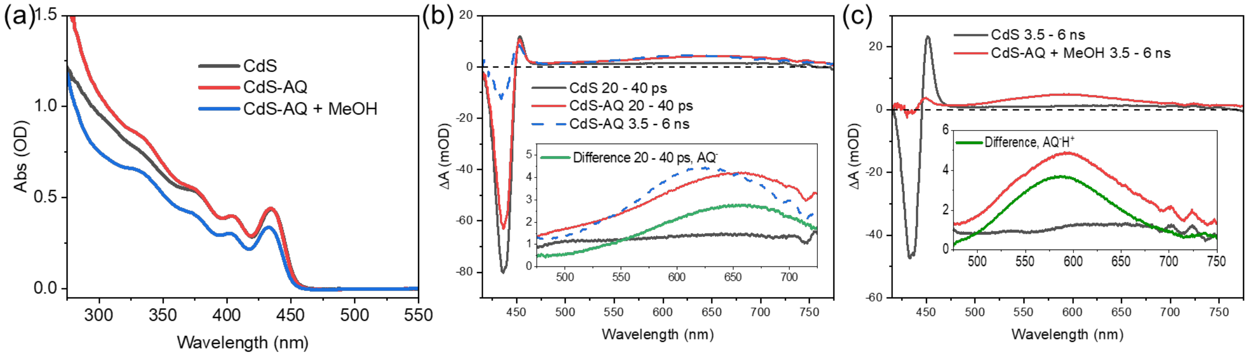

Figure S7. (a) UV-vis absorption of CdS QD (black), CdS-AQ complex (red), and CdSe-AQ complex with 5 % methanol (CdS-AQ + MeOH, blue). (b) TA spectra of CdS QD averaged from 20 ps to 40 ps (black solid curve), CdS-AQ averaged from 20 to 40 ps (red solid curve), and CdS-AQ averaged from 3.5 ns to 6 ns (blue dashed curve). Inset: same spectra zoomed-in from 475 nm to 725 nm. The green solid curve is the difference between the TA spectrum of CdS and CdS-AQ averaged from 20 ps to 40 ps, representing the spectrum of AQ^-. (c) TA spectra of CdS QD averaged from 3.5 ns to 6 ns (black) and CdS-AQ with methanol averaged from 3.5 ns to 6 ns (red). Inset: same spectra zoomed-in from 475 nm to 750 nm. The green curve is the difference between the two spectra, representing the spectrum of AQ^-H^+.

To obtain the reference spectrum of AQ^- and AQ^-H^+, we investigated the charge separated state spectrum of a CdS QD-AQ complex with and without extra proton sources. The CdS QDs used here show the first exciton transition at 435 nm (Figure S7a), far away from the expected AQ^- and AQ^-H^+ spectrum. As a result, in the charge separated state, the AQ^- or AQ^-H^+ absorption line shape should be less impacted by the Stark effect and the XB.

The CdS QDs were synthesized following literature.[5] AQ molecule powder was added into the CdS hexane solution, followed by 10 minutes of sonication to facilitate the adsorption. The mixture was then filtered to remove undissolved AQ molecules. Since AQ is not soluble in hexane by itself, all dissolved AQ molecules are believed to adsorb on the QD surface. Figure S7a shows the absorption spectrum of the filtered CdS-AQ complex solution, which shows extra absorption at < 375 nm compared to pure CdS QD as the evidence of successful loading AQ onto the CdS QDs. To introduce extra proton source into the CdS QD-AQ system, 5 % methanol was added. Our preliminary attempt showed that other organic acids, such as 3-mercaptopropionic acid, cannot maintain the colloidal stability of the QDs. Figure S7a shows that adding methanol does not introduce any spectral changes.

To prepare the reduced AQ or the charge separated state, the CdS QD-AQ samples were pumped at 400 nm. When compared to bare QD, at the delay times from 20 ps to 40 ps (Figure S7b), the CdS QD-AQ sample shows less XB at 435 nm and more PA at > 475 nm, consistent with ET from CdS to AQ. The extra PA in CdS-AQ at > 475 nm is thus regarded as the AQ^- absorption spectrum, whose peak centers at 655 nm (difference spectrum between CdS and CdS-AQ averaged from 20 to 40 ps, Figure S7b, inset).

In the presence of methanol, the sample shows similar ET (Figure S7c). At long delay time, from 3.5 ns to 6 ns, when proton transfer is finished, the CdS QD-AQ + MeOH sample shows an extra PA peak centered at 585 nm (difference spectrum between CdS and CdS-AQ + MeOH averaged from 3.5 ns to 6 ns, Figure S7c, inset). This extra PA peak is regarded as the AQ^-H^+ absorption spectrum. Note that since the QD concentration is different in the CdS-AQ + MeOH (Figure S7a), the CdS PA contribution from 475 nm to 750 nm is first scaled according to the absorptance at excitation (400 nm) and then subtracted from the PA of CdS-AQ + MeOH to obtain the AQ^-H^+ reference spectrum.

It is interesting to note that without extra proton source, at long delay time (from 3.5 to 6 ns), the AQ^- absorption still shows a blue shift from 655 nm to 620 nm (Figure S7b, inset, blue

dashed curve). This blue shift is probably due to incomplete protonation with the protons offered by the carboxylic acid groups in the AQ molecule itself.

S6.5 Linear regression fitting of the TA spectra of InP@ZnSe-AQ and InP@ZnSe:Cu-AQ.

The TA spectra of the QD-AQ samples are then fitted to the linear combination of the spectra of three excited state components using the linear regression fitting method,[3] as shown in equation S1, where $A_{QD-AQ}(\lambda, t)$ is the TA signal as a function of wavelength, λ, and delay time, t, $N(t)$ and $S(\lambda)$ are the relative population as a function of t and base spectrum as a function of λ, respectively. The fitting was conducted in the software MATLAB.

$$A_{QD-AQ}(\lambda, t) = N_{QD^*-AQ}(t) \cdot S_{QD^*-AQ}(\lambda) + N_{QD^+-AQ^-}(t) \cdot S_{QD^+-AQ^-}(\lambda) + N_{QD^+-AQ^-H^+}(t) \cdot S_{QD^+-AQ^-H^+}(\lambda) \qquad (S1)$$

The fitting results are shown in Figure S8. The fitted spectra match well with the experimental spectra, validating the analysis of the contributing components in Figure S6. The fitted $N(t)$ of different components are normalized and plotted in Figure 4d and Figure 4f for InP@ZnSe-AQ and InP@ZnSe:Cu-AQ, respectively.

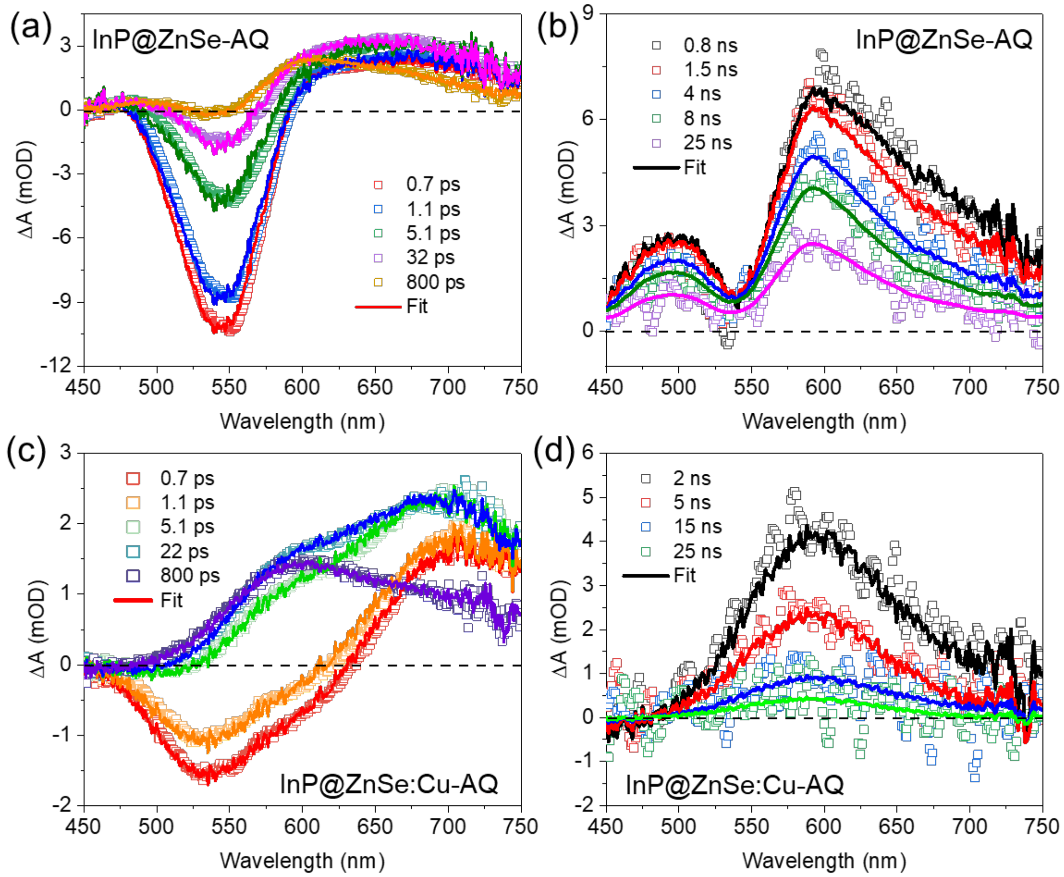

Figure S8. Spectra fitting results. (a, b) Comparison between the experimental TA spectra (colored open squares) and the fitted TA spectra (colored solid lines) in InP@ZnSe-AQ in ps (a) and ns (b)

delay time window. (c, d) Comparison between the experimental TA spectra (colored open squares) and the fitted TA spectra (colored solid lines) in InP@ZnSe:Cu-AQ in ps (c) and ns (d) delay time window.

S6.6 Scaling the QD*-AQ population according to the measured initial amplitude loss.

The population of QD*-AQ (N_{QD^*-AQ}) is not normalized to 1. Instead, the QD*-AQ population is scaled according to the initial amplitude loss of the XB compared to pure QD. For example, in InP@ZnSe-AQ, at 550 nm where the XB signal or the QD*-AQ component dominates, the maximum XB amplitude is -11 mOD at 0.3 ps, while in InP@ZnSe, the maximum amplitude is -15 mOD at <10 ps (Figure S9a). Thus, the maximum of the fitted N_{QD^*-AQ} in InP@ZnSe-AQ is scaled to be 11/15 = 0.73. Similarly, according to Figure S9b, the maximum of the fitted N_{QDCu^*-AQ} in InP@ZnSe:Cu-AQ is scaled to be 0.67/1.78 = 0.38.

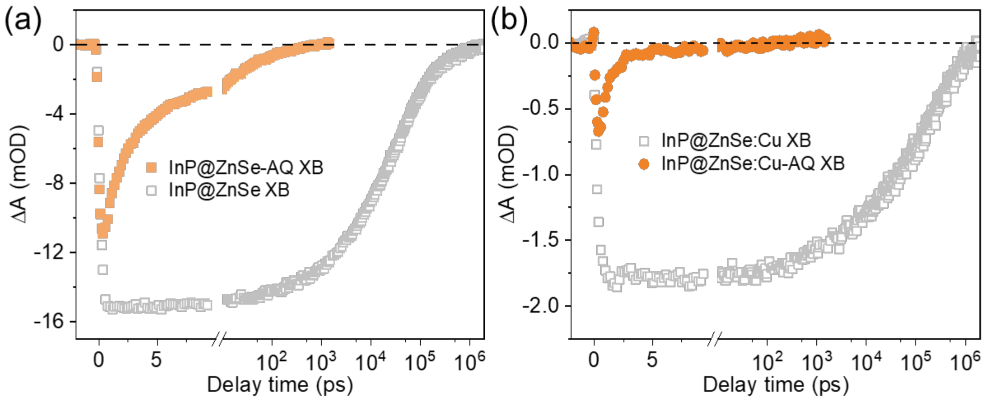

Figure S9. (a) TA kinetics at 550 nm in InP@ZnSe-AQ (orange solid squares) and InP@ZnSe (gray open squares). At 550 nm, the TA signal is dominated by the QD*-AQ component or the XB feature, so the amplitude reflects the relative population of QD*-AQ and the initial amplitude loss percentage reflects the ultrafast ET. Same analysis applies to the TA kinetics at 500 nm in InP@ZnSe:Cu-AQ (orange dots) and InP@ZnSe:Cu (gray open squares) in panel (b).

S6.7 Compare the QD$^+$-AQ$^-$ and QDCu$^+$-AQ$^-$ kinetics.

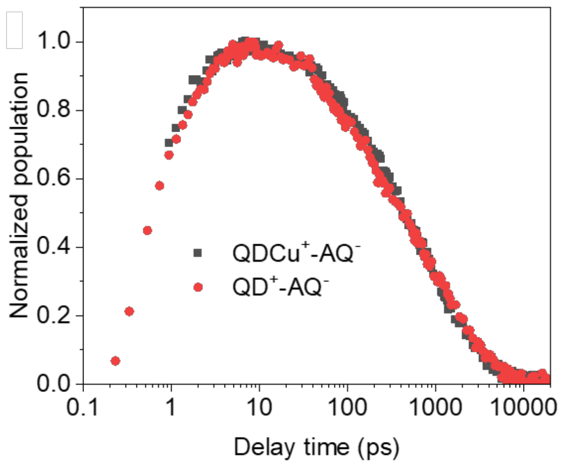

Figure S10. Comparison of $N_{QD^+-AQ^-}(t)$ and $N_{QDCu^+-AQ^-}(t)$ resulted from linear regression fitting. The decay of QD$^+$-AQ$^-$ and QDCu$^+$-AQ$^-$ shows no dependence on Cu doping.

S7. TA kinetics fitting in QD(Cu)-AQ samples.

To study the ET and CR rate constant in the QD(Cu)-AQ samples, the kinetics $N_{QD(Cu)^*-AQ}(t)$, $N_{QD(Cu)^+-AQ^-}(t)$, and $N_{QD(Cu)^+-AQ^-H^+}(t)$ are fit to the model in Figure 4c. Figure S11a shows the same kinetics model with labeled rate constants.

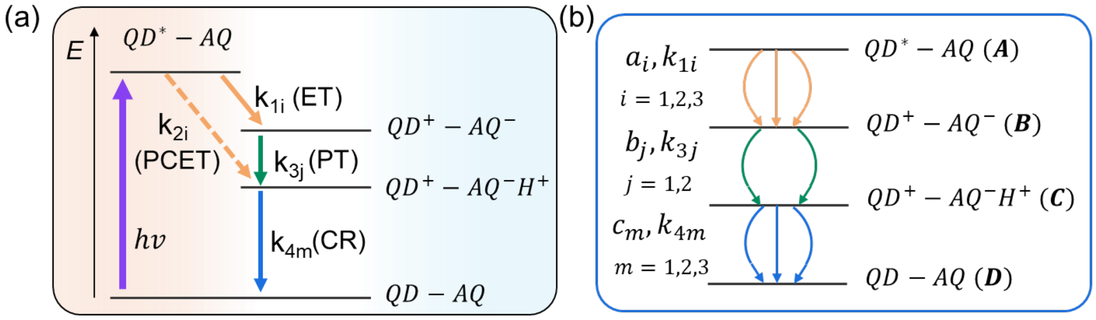

Figure S11. (a) Kinetics model with labeled rate constants. The ET and PCET rate constants are labeled as k_{1i} and k_{2i}, respectively. Proton transfer (PT) rate constant is k_{3j} and the charge recombination (CR) rate constant is k_{4m}. (b) Same kinetics model in (a) with details of relative amplitude (a_i, b_j, c_m) and rate constant (k_{1i}, k_{3j}, k_{4m}) for the decay of each excited state. The subpopulations of each state are independent. In this model, there are $i \times j \times m = 18$ decay pathways from QD*-AQ to QD-AQ. k_{2i} is omitted for simplicity.

Specifically, in InP@ZnSe-AQ, the photogenerated QD*-AQ depopulates through ET (rate constant k_1) and PCET (rate constant k_2):

$$\frac{dN_{QD^*-AQ}}{dt} = -(k_1 + k_2)N_{QD^*-AQ} \qquad (S2)$$

So the QD*-AQ kinetics can be fit to equation S3,

$$N_{QD^*-AQ}(t) = N_0\left(\sum_{i=1}^{3} a_i e^{-(k_{1i}+k_{2i})t} - e^{-k_0 t}\right) \qquad (S3)$$

where N_0 is the total population of photoexcited QD*-AQ and is fixed as 1. k_0 is the QD*-AQ formation rate constant or the hot exciton cooling rate constant. k_0 is obtained by fitting the pure QD data and fixed in equation S3. Three subpopulations are found necessary to fit the QD*-AQ decay kinetics, probably due to three different adsorption geometries. It has been shown in literature that in QD-molecule complexes, different molecule adsorption geometries on QD surface can result in different electronic coupling and thus different electron transfer or energy transfer rate constant. For example, Wu et al. showed different electronic coupling in QD-to-anthracene energy transfer as a result of different anthracene adsorption geometries.[6] As for the specific adsorption geometry of AQ, Cossairt et al. discussed two pathways of anthraquinone adsorption onto QD surface: direct adsorption or replacement with originally weakly bound ligands.[2] Our previous work also demonstrated different adsorption geometries of $Re(4,4'-R_2-bpy)(CO)_3Cl$ (bpy = bipyridine; R = COOH) (ReC0A) on CdP QDs: high ReC0A concentration prefers binding with single carboxyl group, low ReC0A concentration prefers binding with both carboxyl groups.[7] Herein, it may be proposed that the AQ molecules also adsorb onto QD surface by different geometries: binding with single carboxyl group or with both carboxyl groups. Moreover, the tilt angle of surface AQ molecules may also affect the electronic coupling.

QD^+-AQ^- is formed through ET and decays through PT (rate constant k_3):

$$\frac{dN_{QD^+-AQ^-}}{dt} = k_1 N_{QD^*-AQ} - k_3 N_{QD^+-AQ^-} \qquad (S4)$$

Plugging equation S3 into equation S4 leads to the fitting function of $N_{QD^+-AQ^-}(t)$:

$$N_{QD^+-AQ^-}(t) = N_0 a_0 \sum_{i=1}^{3}\sum_{j=1}^{2} \frac{a_i b_j k_{1i}}{k_{3j} - k_{1i} - k_{2i}}\left(e^{-(k_{1i}+k_{2i})t} - e^{-k_{3j}t}\right) \qquad (S5)$$

In equation S5, a_0 is the scaling factor to account for the normalization of $N_{QD^+-AQ^-}$ in Figure 4d. The proton transfer is fit to two exponentials with b_j and k_{3j} as the relative amplitude and rate constant, respectively.

Similarly, QD^+-AQ^-H^+ is formed from both PCET and PT and decays through CR (rate constant k_4):

$$\frac{dN_{QD^+-AQ^-H^+}}{dt} = k_2 N_{QD^*-AQ} + k_3 N_{QD^+-AQ^-} - k_4 N_{QD^+-AQ^-H^+} \qquad (S6)$$

Plugging equation S3 and equation S5 into equation S6 leads to the fitting function of $N_{QD^+-AQ^-H^+}(t)$:

$$N_{QD^+-AQ^-H^+}(t) = N_0 a_0 \sum_{i=1}^{3}\sum_{j=1}^{2}\sum_{m=1}^{3} a_i b_j c_m \left[\frac{k_{2i} + \frac{k_{3j}k_{1i}}{k_{3j}-k_{1i}-k_{2i}}}{k_{4m}-k_{1i}-k_{2i}}\left(e^{-(k_{1i}+k_{2i})t} - e^{-k_{4m}t}\right) - \frac{\frac{k_{3j}k_{1i}}{k_{3j}-k_{1i}-k_{2i}}}{k_{4m}-k_{3j}}\left(e^{-k_{3j}t} - e^{-k_{4m}t}\right)\right] \qquad (S7)$$

In equation S7, the CR is fit to three exponentials with c_m and k_{4m} as the relative amplitude and rate constant, respectively. In the above kinetics model, direct CR from QD^+-AQ^- and the intrinsic QD* decay in QD*-AQ are omitted. The same fitting functions are applied to the InP@ZnSe:Cu-AQ data, except that the ET and PCET are fit to two exponentials rather than three exponentials. The InP@ZnSe-AQ data and InP@ZnSe:Cu-AQ data are globally fitted with shared proton transfer parameters (b_j and k_{3j}). The kinetics fitting results are shown in Figure 4d and Figure 4f for InP@ZnSe-AQ and InP@ZnSe:Cu-AQ, respectively. The fitting parameters are summarized in Table S1.

The averaged ET rate constants and CR rate constants are calculated by equation S8 and equation S9, respectively.

$$k_{ET,avg} = \sum_{i=1}^{3} a_i(k_{1i} + k_{2i}) \qquad (S8)$$

$$k_{CR,avg} = \sum_{m=1}^{3} c_m k_{4m} \qquad (S9)$$

It should be noted that as shown in equation S7 and Figure S11b, the relative amplitude of the exponential decays accounting for ET (a_i), PT (b_j), and CR (c_m) are independent with each other. In another word, the subpopulations of the excited state QD*-AQ, QD^+-AQ^-, and QD^+-AQ^--H^+ are independent. These multiple decay pathways in each state were found necessary to fit the kinetics and were tentatively attributed to different local environments of each excited state. On the other hand, the attempt with a simpler model with dependent subpopulations failed to fit the kinetics, which necessitated the more detailed model in Figure S11b.

Table S1. Fitting parameters of ET, PT, and CR kinetics in InP@ZnSe-AQ and InP@ZnSe:Cu-AQ.

	InP@ZnSe-AQ			InP@ZnSe:Cu-AQ		
i/j/m	1	2	3	1	2	3
a_i (%)	55.2 ± 1.4	29.3 ± 1.5	15.5 ± 1.5	79.4 ± 1.5	20.6 ± 1.5	N/A
k_{1i} (ps^{-1})	0.95 ± 0.04	0	0	2.09 ± 0.09	0.24 ± 0.02	N/A
k_{2i} (ps^{-1})	0	0.14 ± 0.01	0.016 ± 0.001	0.036 ± 0.11	0.11 ± 0.01	N/A
b_j (%)	44.7 ± 0.8	55.3 ± 0.8	N/A	44.7 ± 0.8	55.3 ± 0.8	N/A
k_{3j} (ns^{-1})	5.53 ± 0.17	0.52 ± 0.01	N/A	5.53 ± 0.17	0.52 ± 0.01	N/A
c_m (%)	49.9 ± 0.6	35.3 ± 0.5	14.8 ± 0.6	82.4 ± 1.9	14.6 ± 1.6	2.9 ± 1.9
k_{4m} (μs^{-1})	394.7 ± 14.5	27.6 ± 0.8	0.91 ± 0.06	26615 ± 3076	797 ± 28	50 ± 2
$k_{ET,avg}$ (ps^{-1})		0.57 ± 0.03			1.76 ± 0.08	
$k_{CR,avg}$ (ns^{-1})		0.21 ± 0.01			22.1 ± 2.6	

S8. TA study of ET in QD-MV^{2+} complexes.

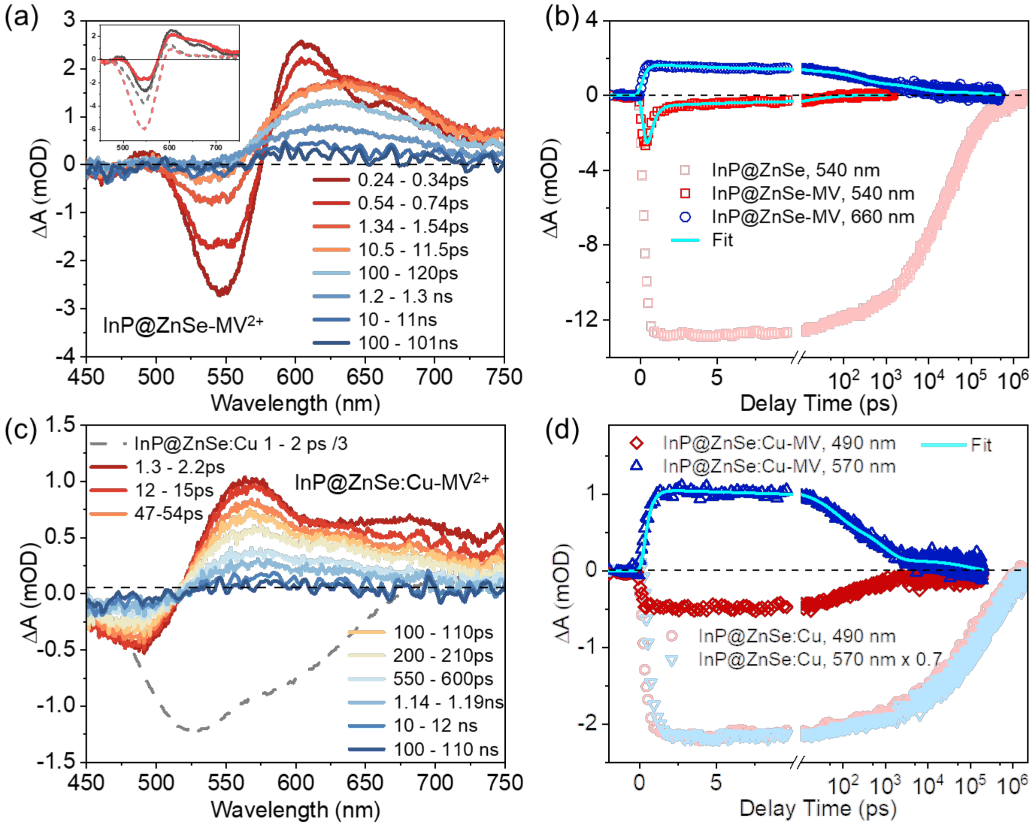

Figure S12. (a) TA spectra of InP@ZnSe-MV^{2+} at indicated delay times upon photoexcitation at 400 nm. Inset: comparison between TA spectra of InP@ZnSe-MV^{2+} (solid curves) and bare InP@ZnSe (dashed curves) at 0.24 – 0.34 ps (black) and 0.54 – 0.74 ps (red). The comparison shows the initial amplitude loss of the XB signal and that the sharp positive peak at 600 nm at early delay time originates from hot exciton rather than reduced MV^{2+} in InP@ZnSe-MV^{2+}. (b) XB (at 540 nm, light red square) kinetics of bare InP@ZnSe QDs and XB (at 540 nm, red square) and PA (at 660 nm, blue circle) kinetics of InP@ZnSe-MV^{2+} complex. (c) TA spectra of InP@ZnSe:Cu-MV^{2+} at indicated delay times. Also shown is the scaled TA spectra of bare InP@ZnSe:Cu QDs at 1 – 2 ps (grey dashed curve). (d) TA kinetics of InP@ZnSe:Cu-MV^{2+} at 490 nm (red diamond) and 570 nm (blue up triangle). Kinetics of InP@ZnSe:Cu at same positions are also shown (490 nm, light red circle; 570 nm, light blue down triangle). The solid cyan curves in (b) and (d) are multiexponential fittings.

Compared to bare InP@ZnSe (Figure 2a) and InP@ZnSe:Cu (Figure 2b) QDs, Figure S12 shows that addition of MV^{2+} causes a much faster decay of XB in both quantum dots, or an initial amplitude loss, which is accompanied by the simultaneous formation of a new absorption peak at > 550 nm. This new feature agrees with the literature reported absorption of reduced MV^{2+} ($MV^{+\bullet}$),[8] which thus confirmed the electron transfer from QDs to the MV^{2+}molecules. The decay of the $MV^{+\bullet}$ signals can then be traced to follow the recombination kinetics between the $MV^{+\bullet}$ and the hole left in the QDs.

In InP@ZnSe-MV^{2+}, the XB at 540 nm shows initial amplitude loss and fast decay (Figure S12b) compared to the XB in bare QDs due to ET to MV^{2+}. The ET kinetics is then extracted by fitting this XB decay to a multi-exponential decay function. In addition, the $MV^{+\bullet}$ PA signal at 660 nm also shows a fast growth, which is globally fitted with the XB decay as they both reflect the ET process. The decay part of this $MV^{+\bullet}$ PA signal kinetics is used to follow the charge recombination. The fitting functions to the XB and $MV^{+\bullet}$ PA kinetics are given in equation S10 and equation S11, respectively.

$$A_{XB}(t) = A_{XB,0}\left(\sum_{i=1}^{3} a_{ET,i} e^{-\frac{t}{\tau_{ET,i}}} - e^{-\frac{t}{\tau_0}}\right) \qquad (S10)$$

$$A_{MV^{+\cdot}}(t) = A_{MV}\left(-\sum_{i=1}^{3} a_{ET,i} e^{-\frac{t}{\tau_{ET,i}}} + \sum_{i=1}^{4} a_{CR,i} e^{-\frac{t}{\tau_{CR,i}}}\right) \qquad (S11)$$

In equation S10, the amplitude $A_{XB,0}$ and the formation time constant τ_0 are fixed as the values in bare QD, so that the initial amplitude loss can be accounted by an ultrafast ET step. $a_{ET,i}$ ($a_{CR,i}$) and $\tau_{ET,i}$ ($\tau_{CR,i}$) are the relative amplitude and time constant of each exponential term in the multiexponential fitting of ET (CR), respectively.

The average rate constants are calculated according to equation S12.

$$k_{avg} = \sum_{i} a_i \frac{1}{\tau_i} \qquad (S12)$$

In InP@ZnSe:Cu-MV^{2+}, the charge-separated (CS) state generated by ET with electron in $MV^{+\bullet}$ and hole in the QD shows a broad derivative feature (Figure S12c). The instantaneous formation of the CS state indicates ultrafast ET to MV^{2+}. The fast decay of the CS state signal (both at 490 nm and 570 nm, Figure S12d) compared to the slow decay of bare QD at the same positions indicates no remaining STE in the presence of MV^{2+} and a shorter lifetime of the CS state. Note that the negative TA signal at 490 nm does not show fast decay component as observed in Figure S12b, which may result from the overlap between the XB decay and the CS state signal (negative part) growth. The ET and charge recombination lifetimes are then obtained by fitting the growth and decay of the $MV^{+\bullet}$ PA signal at 570 nm, respectively, using equation S11. It was found that the ET part, or the growth of the $MV^{+\bullet}$ PA signal can be fit by a single exponential function, while a three-exponential decay function is enough to fit the CR part.

The fitting parameters and the calculated rate constants are listed in Table S2. The fitting curves are shown in Figure S12b, Figure S12d. Note that in Table S2 and Table 1, we compared the rate constant corresponding to the CR half-lifetime ($k_{CR,1/2}$) instead of the amplitude-weighted average rate constant ($k_{CR,avg}$) in InP@ZnSe-MV^{2+} and InP@ZnSe:Cu-MV^{2+}. This is because the calculated $k_{CR,1/2}$ is consistent with the observed faster CR in InP@ZnSe:Cu-MV^{2+}, as clearly shown below in Figure S13b. On the other hand, the amplitude-weighted average CR rate constant ($k_{CR,avg}$) will be biased by the small component with fast decay ($a_{CR,1}$ and $\tau_{CR,1}$).

Table S2. Fitting parameters of ET and charge recombination kinetics in InP@ZnSe-MV^{2+} and InP@ZnSe:Cu-MV^{2+}.

	InP@ZnSe-MV^{2+}	InP@ZnSe:Cu-MV^{2+}
$a_{ET,1}$/%	95.5 ± 0.6	——
$\tau_{ET,1}$/ps	0.31 ± 0.01	0.37 ± 0.04
$a_{ET,2}$/%	2.3 ± 2.3	——
$\tau_{ET,2}$/ps	8.8 ± 10.5	——
$a_{ET,3}$/%	2.2 ± 2.3	
$\tau_{ET,3}$/ps	41.3 ± 35.9	
$a_{CR,1}$/%	23.4 ± 2.9	
$\tau_{CR,1}$/ps	6.3 ± 1.8	
$a_{CR,2}$/%	38.4 ± 2.6	40.3 ± 2.9
$\tau_{CR,2}$/ps	234 ± 40	65.1 ± 9.0
$a_{CR,3}$/%	27.1 ± 2.2	47.0 ± 2.9
$\tau_{CR,3}$/ns	5.83 ± 0.92	0.88 ± 0.10
$a_{CR,4}$/%	11.1 ± 2.9	12.6 ± 2.9
$\tau_{CR,4}$/ns	347 ± 67	87.5 ± 9.4
$k_{ET,avg}$/ps^{-1}	3.12 ± 0.07	2.72 ± 0.30
$k_{CR,1/2}$/ns^{-1}	2.72 ± 0.32	4.08 ± 0.75

S9. Discussion on charge transfer rate constants and efficiencies.

S9.1 Compare electron transfer and charge recombination kinetics.

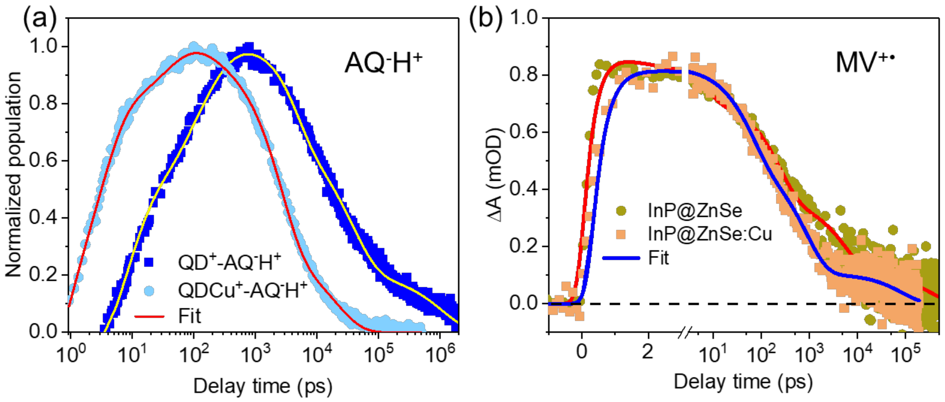

Figure S13. Comparison of kinetics of the reduced electron acceptor $AQ^{-}H^{+}$ (a) and $MV^{+\bullet}$ (b). The amplitudes are scaled for better comparison.

In Figure S13, the formation and decay kinetics of electron acceptors with and without Cu doping are compared. It is clear that the charge recombination is faster with Cu doping for both acceptors.

S9.2 Charge transfer efficiencies.

Table S3. Charge transfer and charge separation efficiencies.

Efficiency/%		InP@ZnSe	InP@ZnSe:Cu
Hole transfer	AA	96.8 ± 0.1	70.6 ± 0.3
	Methanol	37.3 ± 0.8	65.0 ± 0.3
Electron transfer	AQ	99.9 ± 0.0	99.9 ± 0.0
	MV^{2+}	99.9 ± 0.0	99.9 ± 0.0
Charge separation	AA + AQ	74.9 ± 0.9	0.03 ± 0.00
	AA + MV^{2+}	18.8 ± 1.8	0.16 ± 0.03
	Methanol + AQ	5.5 ± 0.2	0.02 ± 0.00
	Methanol + MV^{2+}	0.45 ± 0.05	0.12 ± 0.02

The charge transfer efficiencies, $\eta_{HT(ET)}$, are calculated through equation S13, where $k_{HT(ET)}$ is the hole (electron) transfer rate constant, and $\tau_{exciton}$ is the free exciton or self-trapped exciton lifetime as given in Table 1.

$$\eta_{HT(ET)} = \frac{k_{HT(ET)}}{k_{HT(ET)} + \frac{1}{\tau_{exciton}}} \times 100\% \qquad (S13)$$

For hole transfer to AA, the efficiency is higher for undoped QD due to the faster hole transfer. For hole transfer to methanol, the doped QD shows higher efficiency because the self-trapped exciton lifetime is much longer than the hole transfer lifetime. In the undoped QD, the free exciton lifetime is shorter than the hole transfer lifetime, thus showing a lower hole transfer efficiency.

The electron transfer efficiency is close to unity for all QD-acceptor complexes, which may be caused by the delocalization of the conduction band electron as discussed in the main text.

In the following, we examined the charge separation efficiency, η_{CS}, when the studied electron and hole acceptors are used. Charge separation is defined by the case when both electron and hole are extracted by electron or hole acceptors, respectively. Given the ultrafast electron transfer, the charge separation efficiency is assumed to only depend on the competition between hole extraction and charge recombination after electron transfer, as shown in equation S14.

$$\eta_{CS} = \frac{k_{HT}}{k_{HT} + k_{CR}} \times 100\% \qquad (S14)$$

The calculated η_{CS} (Table S3) are much smaller in doped QDs than in undoped QDs, as a result of the smaller k_{HT} and larger k_{CR} in the doped QDs.

S9.3 No dependence of electron transfer and charge recombination on Cu: In ratio.

The charge transfer kinetics are investigated as a function of the Cu: In ratio. Different amounts of Cu precursor were added during the QD synthesis, resulting in QDs with 7 Cu atoms, 10 Cu atoms (used in the main text), and 18 Cu atoms per QD. The UV-vis absorption spectra of these QDs are shown in Figure S14a. Higher Cu: In ratio leads to larger amplitude of the self-trapped exciton transition. The charge transfer kinetics were examined in the QD-MV^{2+} complexes. Figure S14b shows the rise and decay kinetics of $MV^{+\bullet}$ generated by electron transfer from the QDs to MV^{2+} in the transient absorption measurements. The $MV^{+\bullet}$ kinetics are almost the same for different amounts of Cu dopants per QD. The same electron transfer kinetics (rise of the $MV^{+\bullet}$ signal) may be explained by the delocalized electron wavefunction discussed in the main text. The same charge recombination kinetics (decay of the $MV^{+\bullet}$ signal) may originate from the same Cu dopant energy level and the same radial location of the dopant (between the first and second layer of ZnSe shell), independent on the amount of Cu dopant per QD.

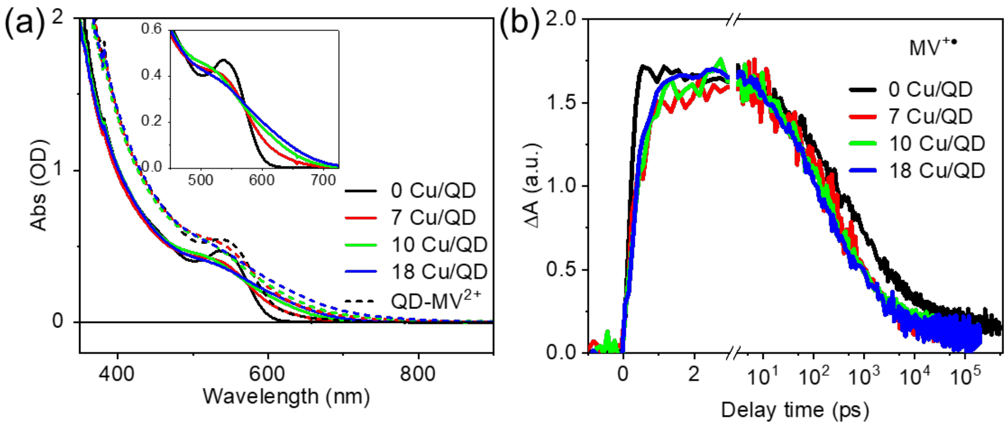

Figure S14. (a) UV-vis absorption spectra (solid curves) of undoped InP@ZnSe QD (0 Cu/QD, black), Cu-doped QDs with 7 Cu atoms per QD (7 Cu/QD, red), 10 Cu atoms per QD (10 Cu/QD, green), and 18 Cu atoms per QD (18 Cu/QD, blue). The short-dashed curves represent the spectra of the corresponding QD-MV^{2+} complexes. Similar to Figure S4b, the additional absorption after adding MV^{2+} is due to the Relay scattering. Inset: zoomed-in view of the absorption spectra from 450 nm to 725 nm. (b) Transient absorption kinetics of $MV^{+\bullet}$ in the QD-MV^{2+} samples with different amount of Cu atoms per QD after 400 nm excitation. The kinetics are scaled to be the same at 10 ps for better comparison.

S10. Calculations of energy levels and surface charge densities.

S10.1 Calculations of reorganization energies.

To determine the value of reorganization energy, we use the additive approximation

$$\lambda_{total} = \lambda_{QD} + \lambda_{Cu} + \lambda_{molecule} + \lambda_{solvent} \tag{S15}$$

where each of the components is considered an independent quantity. The contribution from the undoped QD is taken to be zero, i.e. $\lambda_{QD} = 0$, as is usually done, owing to the delocalized nature of the electron/hole wavefunction. However, the Cu-doped QD is expected to have a non-zero value for reorganization energy which arises from local changes in the lattice at the site of Cu^{+}. Presently we treat it as a parameter and vary λ_{Cu} on the [0,300] meV range (see below). The molecular component may be calculated exactly by measuring the geometry-induced energy change of the molecule after the removal of an electron/hole. The solvent component depends on the electron/hole donor and acceptor sizes and solvent properties and is calculated using the following equation,[9]

$$\lambda_{\text{solvent}} = \frac{1}{2}\left(\frac{1}{2R_{\text{QD}}} + \frac{1}{L} - \frac{2}{2R_{\text{QD}} + L}\right)\left(\frac{1}{\varepsilon_\infty} - \frac{1}{\varepsilon}\right) \tag{S16}$$

where R_{QD} is QD's radius (1.9 nm), L is the size of the molecule (taken to be the longest atom-atom distance), ε_∞ is the optical refractive index and ε is the dielectric constant, 1.375 and 1.89 for hexane, respectively. Table S4 summarizes these calculations.

Table S4. The total and individual components of the reorganization energy (in meV) defined in Eq. S15. The level of theory used for calculating the molecular component is B3LYP/6-31++G(d,p).

	molecule = AA $L = 7$ Å	molecule = methanol $L = 2.8$ Å	molecule = AQ $L = 11$ Å	molecule = MV^{2+} $L = 11$ Å
λ_{QD}	0	0	0	0
λ_{Cu}	0 ~ 300	0 ~ 300	0 ~ 300	0 ~ 300
$\lambda_{molecule}$	313	173	212	300
$\lambda_{solvent}$	178	477	109	109
λ_{total}	491 ~ 791	650 ~ 950	321 ~ 621	409 ~ 709

S10.2 Calculations of electron and hole energy levels and surface charge densities.

In the single-band effective mass approximation (EMA), employed here, the electron and hole wave functions and energies are calculated by solving coupled Schrodinger-Poisson equations discretized on a grid using a variable-mass DVR,[10-12]

$$\left\{-\frac{\hbar^2}{2}\nabla\cdot[\frac{1}{m_e^*}\nabla]+V_e(\mathbf{r})+\frac{1}{2}\Phi_e^{\mathrm{ind}}(\mathbf{r})+\Phi_h(\mathbf{r})\right\}1S_e(\mathbf{r})=E_e1S_e(\mathbf{r}), \quad \text{(S-17a)}$$

$$\nabla\cdot\boldsymbol{\varepsilon}_{\mathbf{r}}\nabla\boldsymbol{\Phi}_{\boldsymbol{e}}(\mathbf{r})=\mathbf{4}\boldsymbol{\pi}\left|\mathbf{1}\boldsymbol{S}_{\boldsymbol{e}}(\mathbf{r})\right|^2, \quad \text{(S-17b)}$$

for the electron in $1S_e$, and a corresponding one for the hole in $1S_h$ orbital

$$\left\{-\frac{\hbar^2}{2}\nabla\cdot[\frac{1}{m_h^*}\nabla]+V_h(\mathbf{r})+\frac{1}{2}\Phi_h^{\mathrm{ind}}(\mathbf{r})+\Phi_e(\mathbf{r})\right\}1S_h(\mathbf{r})=E_h1S_h(\mathbf{r}), \quad \text{(S-18a)}$$

$$\nabla\cdot\boldsymbol{\varepsilon}_{\mathbf{r}}\nabla\boldsymbol{\Phi}_{\boldsymbol{h}}(\mathbf{r})=-\mathbf{4}\boldsymbol{\pi}\left|\mathbf{1}\boldsymbol{S}_{\boldsymbol{h}}(\mathbf{r})\right|^2, \quad \text{(S-18b)}$$

although any pair of orbitals is permitted to be occupied. The parameters entering these equations are described in Table S5. The grid spacing parameter used in the calculations is 2 bohr. The curly brackets in S-17a and S-18a contain, respectively, the effective mass kinetic energy terms, the band potentials for electron and hole, the electrostatic source potentials due to hole and electron, and the self-induced electron and hole potentials with the proper factor of ½.[13] The Poisson equations are solved subject to the condition that the inner and outer potentials match at the surface boundary defined by a cutoff radius $R_{cut}=(x_{cut}^2+y_{cut}^2+z_{cut}^2)^{1/2}$ with $\Psi(x_{cut},y_{cut},z_{cut}) = 0$, where Ψ is 1S state particle's wavefunction.[10-11] The cutoff radius was taken to be that of the dot plus 1 nm.

In other words, the wavefunction was allowed to penetrate up to 1 nm outside QD's surface. The induced potentials Φ_e^{ind} , Φ_h^{ind} are derived from the source potentials Φ_e , Φ_h by integrating the induced surface polarization density[14] over all interfacial (InP/ZnSe and ZnSe/hexane) surfaces I,

$$\Phi_e^{ind}(\mathbf{r}) = \frac{1}{4\pi}\sum_I \frac{\varepsilon_{out}^I - \varepsilon_{in}^I}{\varepsilon_{out}^I} \oint \frac{\vec{\nabla}\Phi_e(\mathbf{r})\Big|_{in} \cdot \hat{n}_{out}^{in}(\mathbf{r}_I)}{|\mathbf{r}-\mathbf{r}_I|} dA_I \quad \text{(S-19a)}$$

$$\Phi_h^{ind}(\mathbf{r}) = \frac{1}{4\pi}\sum_I \frac{\varepsilon_{out}^I - \varepsilon_{in}^I}{\varepsilon_{out}^S} \oint \frac{\vec{\nabla}\Phi_h(\mathbf{r})\Big|_{in} \cdot \hat{n}_{out}^{in}(\mathbf{r}_I)}{|\mathbf{r}-\mathbf{r}_I|} dA_I \quad \text{(S-19b)}$$

Table S5. Material parameters used in the EMA calculations

	InP[(a)]	ZnSe[(b)]	hexane
m_e*	0.08	0.16	1
m_h*	0.64	0.75	1
ε_r	12.5	9.2	1.88
V_e	-3.85	-2.86	0
V_h	-5.2	-5.6	-8.4

m_e* and m_h* are electron and hole effective masses in units of m_0; ε_r is the dielectric constant; V_e and V_h are conduction and valence band edges, respectively, in eV. (a) taken from Ref[15] (b) taken from Ref[16].

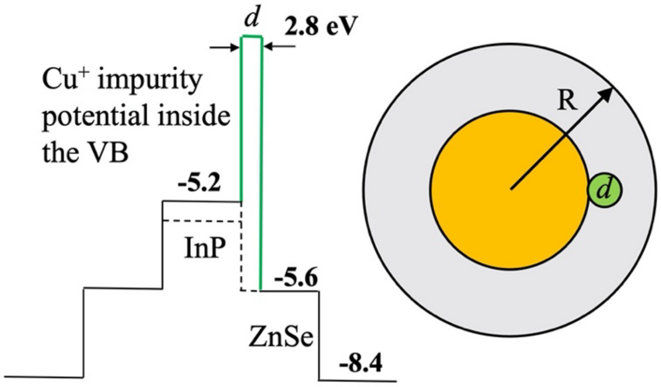

Scheme S-1. Construction of a QD with an embedded impurity. The green area shows location of the impurity (Cu^+ site). The QD radius is R = 1.9 nm; the impurity radius $d/2$ is ~1.5 Å.

In Scheme S-1 we show how the impurity is embedded into the QD. The impurity is treated as a uniform spherical potential of diameter d contained inside the VB of ZnSe shell. The value of the potential is taken from a high-level calculation, B3LYP/LANL2DZ[aug-cc-pVTZ](PCM-1-Hexanol), of the 3d orbital shift from Zn^{2+} in a tetrahedral $ZnSe_4^{6-}$ cluster to Cu^+ in $CuSe_4^{7-}$. The use of a polarizable continuum model with ε = 12.5 approximates the environment's dielectric

constant of the InP Core. The 3d shift was calculated to be 8.384 eV, and is applied to the ZnSe VBM so that the Cu^+ impurity potential in the VB is set at 2.784 eV. (The above electronic structure calculations were performed with the Gaussian 16 program.[17])

The size of the impurity is then estimated by successive adjustment of the diameter *d* until the average exciton wavelength of a doped QD approaches the PL STE emission of 693 nm. For the averaging, we use three samples with impurity positions 1.5, 1.6 and 1.7 nm from the QD center, or 0.2, 0.3 and 0.4 nm from InP core sphere, respectively. This procedure yielded the impurity diameter of d = 2.96 Å, a value comparable to the ZnSe zincblende lattice parameter of ~5.6 Å, or the Zn-Se bond length of ~2.4 Å. In other words, the hole wavefunction is expected to be localized on the 3d orbital of Cu. Figure S15 shows the corresponding wavefunctions.

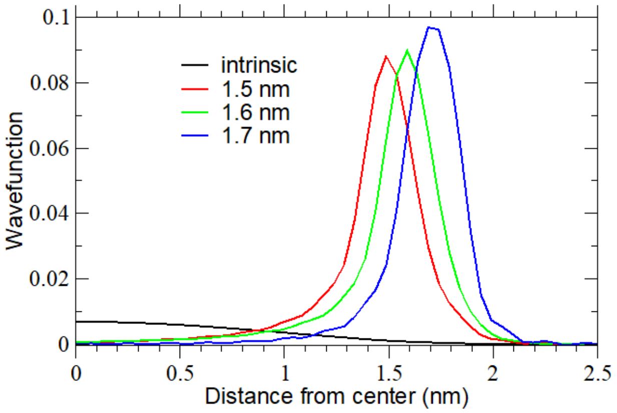

Figure S15. Hole wavefunctions in the intrinsic QD, and with Cu impurities embedded at 1.5, 1.6 and 1.7 nm from QD center. The Shell's inner and outer spheres are at 1.3 and 1.9 nm, respectively.

From the calculated wavefunctions we determine the electron and hole 1S energy levels and the quantum coupling strength H_{12} factor, which is proportional to the hole's surface charge density at the outer shell, averaging these quantities over the shell volume. To shed light on the exciton recombination lifetime, the overlap squared of the hole wavefunction with the electron wavefunction is also calculated.

Table S6 summarizes these results. One can see a strong increase in surface charge density ($|H_{12}|^2$) in the doped QDs and a noticeable increasing trend as the impurity moves farther away from the InP Core. Specifically, the surface hole density increases by 7, 22, and 87 times when the Cu dopant locates at 1.5 nm, 1.6 nm, and 1.7 nm, respectively, compared to the intrinsic QD. In the following discussion of Maruc theory, the enhancement factor 7 is used as the Cu dopants are introduced after the first ZnSe layer and before the second ZnSe layer.

The opposite trend is observed with the electron-hole overlap, which decreases by virtue of hole's strong localization, suggesting a longer lived exciton. The electron energy level stays virtually constant for the intrinsic and doped QDs and independent of Cu^+ position inside the ZnSe shell. The hole energy level shifts deep into the gap region and varies slightly with dopant position.

Using the averaged values, we can determine that the Cu-doped QD hole energy level relative to its level in the intrinsic QD shifts up, i.e., toward the AA, AQ redox levels, by 0.363 eV.

Table S6. Band gap E_0, electron and hole energy levels E_e and E_h, electron-hole Coulomb interaction energy ΔW, hole surface charge density $|H_{12}|^2$, and electron-hole overlap squared $|\langle 1S_e|1S_h\rangle|^2$ for the intrinsic and Cu-doped QDs (with Cu^+ sites location inside the CdSe shell), and their volume averaged values.

	E_0/eV	E_e/eV	E_h/eV	ΔW/eV	$|H_{12}|^2$	$|\langle 1S_e|1S_h\rangle|^2$
intrinsic	2.152	-3.388	-5.089	-0.451	5.76e-4	0.87
1.5 nm	1.732	-3.362	-4.663	-0.431	3.97e-3	0.65e-1
1.6 nm	1.792	-3.358	-4.723	-0.427	1.28e-2	0.61e-1
1.7 nm	1.836	-3.353	-4.769	-0.420	5.02e-2	0.11e-1
average	1.792	-3.360	-4.726	-0.426	2.13e-2	0.48e-1

S10.3 Driving force of charge recombination in InP@ZnSe-AQ.

Scheme 1b shows the charge recombination driving force in QD^+-AQ^- is -1.09 eV. However, the protonation of AQ^- results in the AQ^-H^+ in a lower energy, reducing the net driving force. This driving force change is estimated by the free energy change of the protonation reaction, which can be inferred from the pK_a value of AQ^-H^+. The pK_a of the singly reduced neutral semiquinone radical of various anthraquinone derivatives are reported to be 3.3 ~ 5.4 in aqueous solutions,[18-20] corresponding to a free energy change of -0.195 eV ~ -0.313 eV for the protonation reaction. Thus, adopting this free energy change value, the driving force for the charge recombination in QD^+-AQ^-H^+ is estimated to be -0.78 ~ -0.90 eV. In the following simulation of the rate constant ratios (section S11), the charge recombination driving force is taken as the average value, -0.84 eV.

S11. Effect of driving force, reorganization energy, and dopant density on rate constants

S11.1 Effect of driving force and reorganization energy

Table S7. Parameters used for simulating the rate constants.

	HT		CR	
	AA	Methanol	$AQ^{-}H^{+}$	$MV^{+\bullet}$
$\varphi_{A+/A}$ vs NHE (V)[a]	0.2	0	-0.25	-0.24
$H^2_{DA,\ QDCu}/H^2_{DA,\ QD}$[b]	7			
$\lambda_{total,\ QD}$[c] (eV)	0.49	0.65	0.32	0.41
$\lambda_{total,\ QDCu}$[d] (eV)	0.49 ~ 0.79	0.65 ~ 0.95	0.32 ~ 0.62	0.41 ~ 0.71
ΔG_{QD} (eV)	-0.39	-0.59	-0.84	-0.83
ΔG_{QDCu} (eV)	-0.01	-0.21	-0.46	-0.45

[a]$\varphi_{A+/A}$ is the redox potential of hole acceptors versus NHE. $\varphi_{A+/A}$ of methanol is from reference[21]. $\varphi_{A+/A}$ of $MV^{+\bullet}$ is from reference[22]. [b]The coupling enhancement factor is discussed in section S10.2. [c]The reorganization energy calculation is shown in Table S4. [d]The Cu dopant related reorganization energy λ_{Cu} is varied from 0 to 0.3 eV.

The hole transfer (HT) and charge recombination (CR) rate constants are simulated using the Marcus theory, as shown in equation S20 (same as the equation in the main text). The parameters used for the simulation are given in Table S7. The net driving force for InP@ZnSe:Cu is smaller than that for InP@ZnSe by 0.38 eV due to the hole trapping into Cu dopants, as discussed in Figure 1b. It should be noted that such simulations using Marcus theory can offer qualitative explanation to the observed trend in the charge transfer rate constants, although a quantitative account is difficult due to uncertainties in key parameters (such as coupling strength, driving force, and reorganization energy).

$$k_{QD} = \frac{2\pi}{\hbar} \frac{H^2_{DA,QD}}{\sqrt{4\pi\lambda_{total,QD}k_bT}} exp\left(-\frac{\left(\Delta G_{QD} + \lambda_{total,QD}\right)^2}{4\lambda_{total,QD}k_bT}\right) \quad (Eq. 20-a)$$

$$k_{QDCu} = \frac{2\pi}{\hbar} \frac{H^2_{DA,QDCu}}{\sqrt{4\pi\lambda_{total,QDCu}k_bT}} exp\left(-\frac{\left(\Delta G_{QDCu} + \lambda_{total,QDCu}\right)^2}{4\lambda_{total,QDCu}k_bT}\right) \quad (Eq. 20-b)$$

The Cu dopant impact on charge transfer can be defined by the ratio k_{QDCu}/k_{QD} for each charge transfer reaction. The calculated k_{QDCu}/k_{QD} is shown in Figure S16 as a function of λ_{Cu}. These modeling results indicate that Cu doping slows down HT rates ($\frac{k_{QDCu}}{k_{QD}} < 1$) and accelerates

CR rates ($\frac{k_{QDCu}}{k_{QD}} > 1$). Table S7 shows that both HT to AA and to methanol fall into the Marcus normal region, thus showing slower HT rate constant when the net driving force is reduced by Cu doping. On the other hand, when λ_{Cu} is 0, both CR with $AQ^{-}H^{+}$ and $MV^{+\bullet}$ fall into the inverted region, as discussed in the main text, and reducing the net driving force would increase the CR rate constants. As illustrated in Figure S16, varying the λ_{Cu} negligibly changes the Cu dopant impact on charge transfer.

In addition, the simulation shows that the Cu doping impact on HT to AA is much larger than that on HT to methanol and similarly, Cu doping affects CR with $AQ^{-}H^{+}$ much more than CR with $MV^{+\bullet}$. Both are consistent with the experimental results shown in Table 1. Again, varying the λ_{Cu} does not alter qualitative trends of HT and CR among different acceptors.

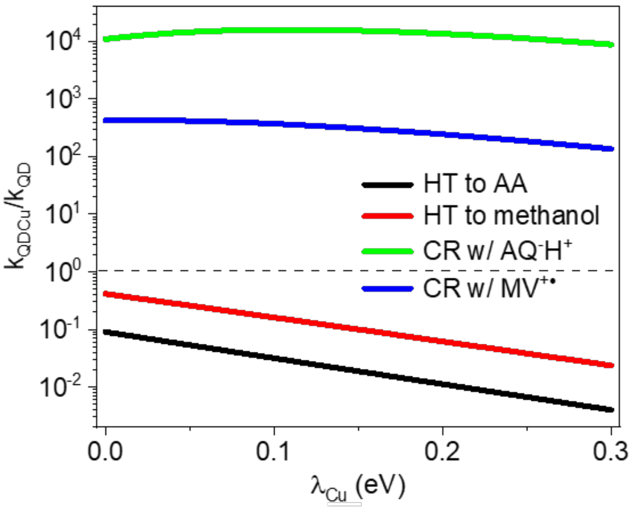

Figure S16. Rate constant ratio between intrinsic QD and Cu-doped QD as a function of λ_{Cu}. The dashed line indicates the ratio of 1.

These different impacts of Cu doping on different acceptors are mainly attributed to the difference in the acceptor reorganization energies according to the Marcus theory. For example, Figure S17 shows that, although the CR with $AQ^{-}H^{+}$ and $MV^{+\bullet}$ show the similar driving force (-0.83 ~ -0.84 eV for intrinsic QD, and -0.45 ~ -0.46 eV for Cu-doped QD), reducing the driving force from -0.84 eV to -0.46 eV introduces much larger change of the rate constant with $AQ^{-}H^{+}$ than with $MV^{+\bullet}$. This is because $MV^{+\bullet}$ has a larger reorganization energy (0.41 eV) than $AQ^{-}H^{+}$ (0.32 eV), which leads to a less sensitive dependence of rate constants on the driving force change. Similar situation occurs for HT to AA and to methanol, where reorganization energy of HT to methanol is larger than that of HT to AA.

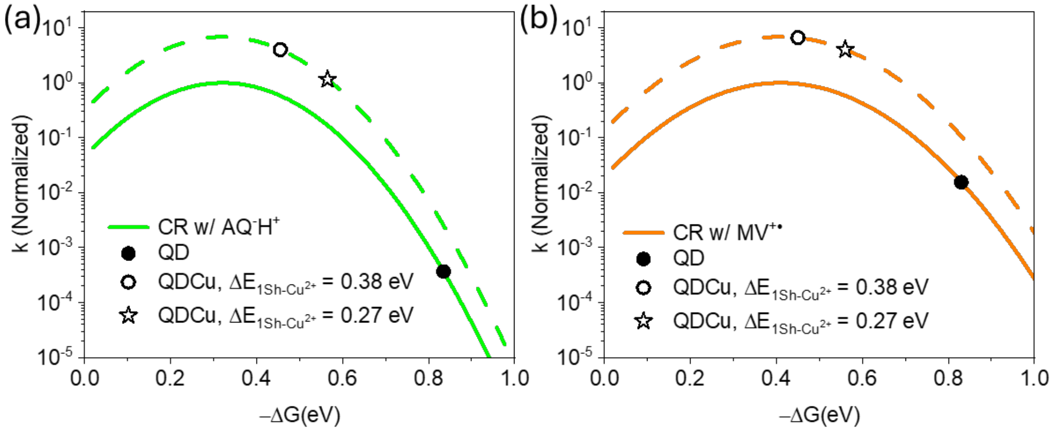

Figure S17. Normalized rate constants as a function of net driving force for CR with $AQ^{-}H^{+}$ (a) and CR with $MV^{+\bullet}$ (b). The solid and dashed curves describe CR in InP@ZnSe (QD) and InP@ZnSe:Cu (QDCu), respectively. In QDCu (dashed curve) the rate constant is enhanced by the coupling term $H_{DA}^2 = 7$. The λ_{Cu} is set as 0 for simplicity. The black dots represent the rate constants for QD. The white dots and stars represent the rate constants for QDCu with the 1Sh-Cu^{2+} energy difference ($\Delta E_{1Sh-Cu^{2+}}$) of 0.38 eV and 0.27 eV, respectively.

We note that the rate constant ratio k_{QDCu}/k_{QD} for both CR reactions in Figure S16 is much larger than the measured ratio. Several factors can contribute to the overestimated k_{QDCu}/k_{QD} obtained from Marcus theory. For example, overestimation of the intrinsic QD driving force and the coupling term H_{DA}^2 can lead to a larger k_{QDCu}/k_{QD}, as can be anticipated from Figure S17.

Specifically, although the Cu dopant can increase the average hole density at the QD surface, as discussed in Figure S15, the electronic coupling for CR reaction can be smaller compared to intrinsic QDs (Figure S18a) in the case where the reduced electron acceptor is on the opposite side of the QD with respect to the Cu dopant (Figure S18c). This reduced electronic coupling may contribute to the smaller experimental k_{QDCu}/k_{QD} than the k_{QDCu}/k_{QD} predicted by the Marcus theory in Figure S16, which used an average electronic coupling enhancement factor of 7. For hole transfer to hole acceptors, the hole in Cu dopant is expected to transfer to a closely located acceptor and will not suffer from the smaller coupling illustrated in Figure S18c. Nevertheless, the impact of the Cu-doping induced electronic coupling changes on charge transfer is overwhelmed by the impact of the driving force changes, as suggested by the observed slower hole transfer, the faster CR, and the Marcus theory analysis.

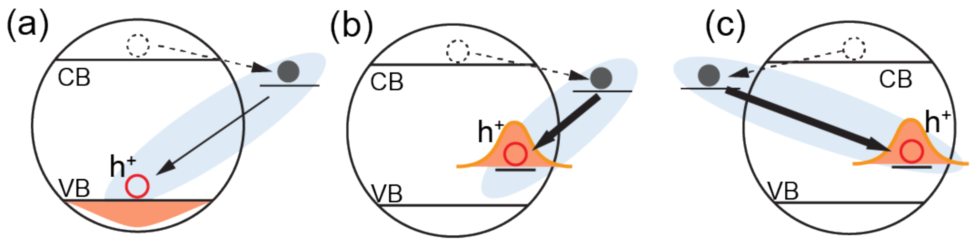

Figure S18. Illustration of the electronic coupling between the hole and the electron in the reduced electron acceptor in (a) intrinsic QD, (b) Cu-doped QD where the acceptor is on the same side as the Cu dopant, and (c) Cu-doped QD where the acceptor is on the opposite side.

In addition, overestimation of the Cu doping induced driving force change (the energy difference between the $1S_h$ state and the Cu^{2+} state, $\Delta E_{1Sh-Cu^{2+}}$) can also lead to a larger k_{QDCu}/k_{QD} . $\Delta E_{1Sh-Cu^{2+}}$ is estimated from the photoluminescence spectrum peak energy differences between InP@ZnSe and InP@ZnSe:Cu (Figure S19a and Figure 1b in manuscript). This energy difference (0.38 eV, Figure S19b and Figure S19c) may contain contributions from the extra Stokes shift in InP@ZnSe:Cu due to lattice reorganization around the Cu dopant upon hole trapping.[23] The other method to estimate $\Delta E_{1Sh-Cu^{2+}}$ is to compare the absorption energy of the $1S_h$-$1S_e$ and Cu-$1S_e$ transitions, as shown in Figure S19d~f. The transient absorption (TA) spectrum of InP@ZnSe at delay time of 1 ns (Figure S19d, blue spectrum) shows bleach peak at 2.28 eV according to the bleach of the $1S_h$-$1S_e$ transition (Figure S19f). The TA spectrum of InP@ZnSe:Cu at 1 ns (Figure S19d, red spectrum) shows the same major bleach at 2.28 eV and a broad shoulder centering around 2.01 eV, which is attributed to the bleach of the Cu-$1S_e$ transition, as shown in Figure S19e. The energy difference of the $1S_h$-$1S_e$ and Cu-$1S_e$ transitions is then estimated to be 0.27 eV (Figure S19e), smaller than the value estimated from the PL spectra. This smaller energy difference renders larger net driving forces for InP@ZnSe:Cu QDs in the studied charge transfer reactions. Figure S17 shows that, using the larger driving forces for InP@ZnSe:Cu (white star), k_{QDCu}/k_{QD} is smaller and closer to the experimental results and can still explain the discrepancy between different acceptors. A more refined modeling would require the knowledge of the exact energy distribution of the Cu dopant state and the $1S_h$ state. Nevertheless, the above analyses demonstrate the observed trend of Cu doping on different acceptors can be rationalized qualitatively by Marcus theory.

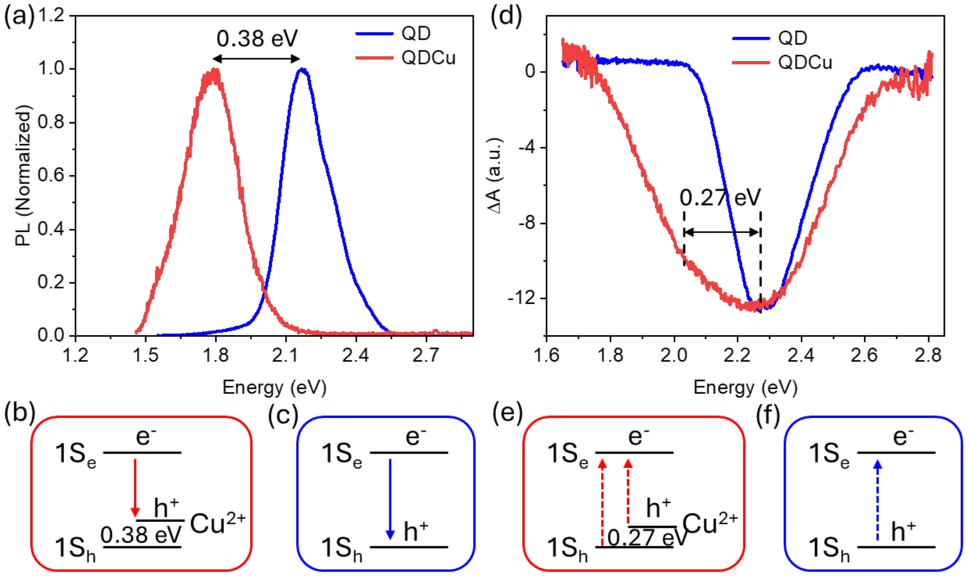

Figure S19. Demonstration of the energy difference between the $1S_h$ state and the Cu^{2+} state. (a) photoluminescence (PL) spectra of InP@ZnSe (QD, blue) and InP@ZnSe:Cu (QDCu, red), showing the peak energy difference of 0.38 eV. Schematics of the PL emission in QDCu (b) and QD (c), attributing the PL energy difference to the $1S_h$ and Cu^{2+} states splitting. (d) Transient absorption (TA) spectrum of QD (blue) and QDCu (red) at 1 ns delay time after 400 nm pump. In QDCu, the shoulder bleach peak position is 0.27 eV lower than that of the main bleach peak. Schematics of the TA bleach signal origins in QDCu (e) and QD (f). The self-trapped exciton in QDCu (e) bleaches both the $1S_h$-$1S_e$ and Cu-$1S_e$ transitions, with the later contributing to the shoulder bleach in the QDCu TA spectrum in (d), while the free exciton in QD (f) only bleaches the $1S_h$-$1S_e$ transition. The energy difference between the $1S_h$-$1S_e$ and Cu-$1S_e$ transitions is 0.27 eV.

S11.2 Effect of dopant density

As an aside we mention that embedding more than one impurity was also considered. The calculations show that placing two impurities centro-symmetrically leads to double-well type of symmetric solutions with two density pockets localized at the impurities. The average surface charge density is nearly identical to the case of a single impurity. On the other hand, placing two impurities asymmetrically leads to formation of a single density pocket, nearly identical to one with the single impurity. Averaging the results over all possible pairs (1.5/1.6 nm, 1.5/1.7 nm, etc) leads to the results very similar to those in Table S6. However, if one considers volume scaling in a random impurity placement, i.e. the R^2 rule, there will be more impurities found at the longer distances away from the center, which means closer to the outer Shell surface. In other words, addition of extra impurities should increase hole's surface charge density and improve its coupling with the acceptor.

References:

1. Xie, R. G.; Peng, X. G., Synthesis of Cu-Doped InP Nanocrystals (d-dots) with ZnSe Diffusion Barrier as Efficient and Color-Tunable NIR Emitters. *Journal of the American Chemical Society* **2009,** *131* (30), 10645-10651.
2. Eagle, F. W.; Harvey, S.; Beck, R.; Li, X.; Gamelin, D. R.; Cossairt, B. M., Enhanced Charge Transfer from Coinage Metal Doped InP Quantum Dots. *ACS Nanosci Au* **2023,** *3* (6), 451-461.
3. Zhu, H.; Song, N.; Lian, T., Controlling Charge Separation and Recombination Rates in CdSe/ZnS Type I Core-Shell Quantum Dots by Shell Thicknesses. *Journal of the American Chemical Society* **2010,** *132* (42), 15038-15045.
4. de Juan, A.; Jaumot, J.; Tauler, R., Multivariate Curve Resolution (MCR). Solving the mixture analysis problem. *Anal. Methods* **2014,** *6* (14), 4964-4976.
5. Wang, J.; Ding, T.; Wu, K., Charge Transfer from n-Doped Nanocrystals: Mimicking Intermediate Events in Multielectron Photocatalysis. *J Am Chem Soc* **2018,** *140* (25), 7791-7794.
6. He, S.; Lai, R.; Jiang, Q.; Han, Y.; Luo, X.; Tian, Y.; Liu, X.; Wu, K., Engineering Sensitized Photon Upconversion Efficiency via Nanocrystal Wavefunction and Molecular Geometry. *Angew Chem Int Ed Engl* **2020,** *59* (40), 17726-17731.
7. Gebre, S. T.; Kiefer, L. M.; Guo, F.; Yang, K. R.; Miller, C.; Liu, Y.; Kubiak, C. P.; Batista, V. S.; Lian, T., Amine Hole Scavengers Facilitate Both Electron and Hole Transfer in a Nanocrystal/Molecular Hybrid Photocatalyst. *J Am Chem Soc* **2023,** *145* (5), 3238-3247.
8. Zhu, H.; Song, N.; Rodriguez-Cordoba, W.; Lian, T., Wave Function Engineering for Efficient Extraction of up to Nineteen Electrons from One CdSe/CdS Quasi-Type II Quantum Dot. *J Am Chem Soc* **2012,** *134* (9), 4250-7.
9. Marcus, R. A., Chemical and electrochemical electron-transfer theory. *Annual review of physical chemistry* **1964,** *15* (1), 155-196.
10. Kaledin, A. L.; Lian, T.; Hill, C. L.; Musaev, D. G., An Infinite Order Discrete Variable Representation of an Effective Mass Hamiltonian: Application to Exciton Wave Functions in Quantum Confined Nanostructures. *Journal of Chemical Theory and Computation* **2014,** *10* (8), 3409-3416.
11. Kaledin, A. L.; Lian, T.; Hill, C. L.; Musaev, D. G., A hybrid quantum mechanical approach: Intimate details of electron transfer between type-I CdSe/ZnS quantum dots and an anthraquinone molecule. *The Journal of Physical Chemistry B* **2015,** *119* (24), 7651-7658.
12. Jia, Y.; Chen, J.; Wu, K.; Kaledin, A.; Musaev, D. G.; Xie, Z.; Lian, T., Enhancing photo-reduction quantum efficiency using quasi-type II core/shell quantum dots. *Chemical Science* **2016,** *7* (7), 4125-4133.
13. Allan, G.; Delerue, C.; Lannoo, M.; Martin, E., Hydrogenic impurity levels, dielectric constant, and Coulomb charging effects in silicon crystallites. *Physical Review B* **1995,** *52* (16), 11982.
14. Jackson, J. D., *Classical electrodynamics*. John Wiley & Sons: 2012.
15. Xiang, X.; Fielden, J.; Rodríguez-Córdoba, W.; Huang, Z.; Zhang, N.; Luo, Z.; Musaev, D. G.; Lian, T.; Hill, C. L., Electron transfer dynamics in semiconductor–chromophore–polyoxometalate catalyst photoanodes. *The Journal of Physical Chemistry C* **2013,** *117* (2), 918-926.
16. Kim, K.; Lee, H.; Ahn, J.; Jeong, S., Highly luminescing multi-shell semiconductor nanocrystals InP/ZnSe/ZnS. *Applied Physics Letters* **2012,** *101* (7).
17. Frisch, M.; Trucks, G.; Schlegel, H.; Scuseria, G.; Robb, M.; Cheeseman, J.; Scalmani, G.; Barone, V.; Petersson, G.; Nakatsuji, H., Gaussian 16 Revision C. 01, 2016. *Gaussian Inc. Wallingford CT* **2016,** *1*, 572.
18. Wightman, R. M.; Cockrell, J. R.; Murray, R. W.; Burnett, J. N.; Jones, S. B., Protonation kinetics and mechanism for 1,8-dihydroxyanthraquinone and anthraquinone anion radicals in dimethylformamide solvent. *Journal of the American Chemical Society* **1976,** *98* (9), 2562-2570.
19. Gamage, R. S. K. A.; McQuillan, A. J.; Peake, B. M., Ultraviolet–visible and electron paramagnetic resonance spectroelectrochemical studies of the reduction products of some anthraquinone sulphonates in aqueous solutions. *J. Chem. Soc., Faraday Trans.* **1991,** *87* (22), 3653-3660.
20. Babaei, A.; Connor, P. A.; McQuillan, A. J.; Umapathy, S., UV-Visible Spectrooelectrochemistry of the Reduction Products of Anthraquinone in Dimethylformamide Solutions: An Advanced Undergraduate Experiment. *Journal of Chemical Education* **1997,** *74* (10), 1200.
21. Wu, K.; Chen, Z.; Lv, H.; Zhu, H.; Hill, C. L.; Lian, T., Hole Removal Rate Limits Photodriven H-2 Generation Efficiency in CdS-Pt and CdSe/CdS-Pt Semiconductor Nanorod-Metal Tip Heterostructures. *Journal of the American Chemical Society* **2014,** *136* (21), 7708-7716.
22. Zhu, H.; Yang, Y.; Hyeon-Deuk, K.; Califano, M.; Song, N.; Wang, Y.; Zhang, W.; Prezhdo, O. V.; Lian, T., Auger-assisted electron transfer from photoexcited semiconductor quantum dots. *Nano Lett* **2014,** *14* (3), 1263-9.

23. Prins, P. T.; Spruijt, D. A. W.; Mangnus, M. J. J.; Rabouw, F. T.; Vanmaekelbergh, D.; de Mello Donega, C.; Geiregat, P., Slow Hole Localization and Fast Electron Cooling in Cu-Doped InP/ZnSe Quantum Dots. *J Phys Chem Lett* **2022**, 9950-9956.
